# Intratumoral Microbiome: Foe or Friend in Reshaping the Tumor Microenvironment Landscape?

**DOI:** 10.3390/cells13151279

**Published:** 2024-07-30

**Authors:** Athina A. Kyriazi, Makrina Karaglani, Sofia Agelaki, Stavroula Baritaki

**Affiliations:** 1 Laboratory of Experimental Oncology, Division of Surgery, School of Medicine, University of Crete, 71500 Heraklion, Greece; molgrad431@edu.biology.uoc.gr; 2 Laboratory of Pharmacology, Department of Medicine, Democritus University of Thrace, 68100 Alexandroupolis, Greece; mkaragla@med.duth.gr; 3 Laboratory of Hygiene and Environmental Protection, Democritus University of Thrace, 68100 Alexandroupolis, Greece; 4 Laboratory of Translational Oncology, School of Medicine, University of Crete, 71500 Heraklion, Greece; agelaki@uoc.gr

**Keywords:** microbiome, cancer, tumor microenvironment, metabolism, immune cells, therapy

## Abstract

The role of the microbiome in cancer and its crosstalk with the tumor microenvironment (TME) has been extensively studied and characterized. An emerging field in the cancer microbiome research is the concept of the intratumoral microbiome, which refers to the microbiome residing within the tumor. This microbiome primarily originates from the local microbiome of the tumor-bearing tissue or from translocating microbiome from distant sites, such as the gut. Despite the increasing number of studies on intratumoral microbiome, it remains unclear whether it is a driver or a bystander of oncogenesis and tumor progression. This review aims to elucidate the intricate role of the intratumoral microbiome in tumor development by exploring its effects on reshaping the multileveled ecosystem in which tumors thrive, the TME. To dissect the complexity and the multitude of layers within the TME, we distinguish six specialized tumor microenvironments, namely, the immune, metabolic, hypoxic, acidic, mechanical and innervated microenvironments. Accordingly, we attempt to decipher the effects of the intratumoral microbiome on each specialized microenvironment and ultimately decode its tumor-promoting or tumor-suppressive impact. Additionally, we portray the intratumoral microbiome as an orchestrator in the tumor milieu, fine-tuning the responses in distinct, specialized microenvironments and remodeling the TME in a multileveled and multifaceted manner.

## 1. Introduction

It has been a longstanding view that cancer is not simply a disease driven solely by transformed tumor cells, but rather that the formation and progression of tumors are heavily dependent on the milieu in which the tumor cells reside. The term “tumor microenvironment (TME)” was coined to describe the tumor milieu, composed of both cell and non-cell components, as well as the complex interplay between them. The concept of TME initially derived by Stephen Paget’s “seed and soil” hypothesis, dated back in 1889, which described the preference of cancer cells to colonize specific organs, with a presumably suitable microenvironment [1]. In the last 50 years, extensive research efforts have shed light on the composition and functionality of the TME, thus extending the initial Paget’s hypothesis for the importance of the microenvironment for both primary and metastatic tumors [2].

The cellular fraction of the TME consists of numerous cell types of different origin, such as immune cell populations, cancer-associated fibroblasts (CAFs), endothelial cells, pericytes and adipocytes. These cells, in turn, secrete a variety of soluble factors, including cytokines, chemokines, adipokines, metabolites, extracellular vesicles and signaling molecules, which constitute a major part of the non-cellular TME component [3]. In addition, newly identified nerve fibers within the TME have added further complexity to the TME composition, along with its functional significance [4]. Apart from the cellular fraction of the TME, the extracellular matrix (ECM) and tumor vasculature are major non-cellular components of the TME that surround malignant and non-malignant cells. In addition, the existence of interstitial fluid also comprises a significant feature of the TME, which functions as a remover of cell wastes in the TME, while it contains cell-secreted soluble factors, along with water-dissolved nutrients and electrolytes needed to be continuously supplied to the tumor and surrounding cells [5]. This fluid is further characterized by increased hydrostatic and colloid osmotic pressures, due to the leaky tumor vasculature and abnormal tumor lymphatics, thus affecting the drug delivery within the TME [6]. 

The interplay between distinct cellular and non-cellular components of the TME is highly complex and it dictates the outcomes of cancer-associated processes, including oncogenesis, tumor progression and metastasis [3]. Notably, the heterogeneity in tumor cell phenotypes within the tumor tissue reshapes the TME composition, while the TME components also influence the tumor evolution, thus establishing a bidirectional relationship between cancer cells and their microenvironment [7]. Given this high abundance and variety of TME components as well as their intricate crosstalk, several TME dissecting and compartmentalization approaches have been emerged over the last ten years, aiming to decipher the underlying cancer cell biology within the tumor milieu [8]. The prevailing approach attempted the compartmentalization of the TME into six specialized sub-microenvironments, including immune, metabolic, hypoxic, acidic, mechanical and innervated microenvironments, all of which contribute to fundamental cancer hallmarks and foster cancer development [9,10].

Recently, mounting evidence from preclinical and clinical studies suggests that the composition of the human microbiome comprises one of the major integral parts and modifiers of TME. The microbiome is characterized as “the second genome of the human body”, as the population of microbial cells within the human body surpasses the number of human cells by approximately tenfold [11]. The human microbiota population consists of bacteria, fungi, archaea, protozoans and viruses, which reside in different body locations, such as the skin, oral cavity, respiratory tract and vagina but primarily in the gut [12,13]. Due to their abundance and omnipresence in the human body, it is not surprising that changes in microbiome composition have been suggested to be implicated in the pathogenesis of many human diseases, including systematic infections; cardiovascular, liver and respiratory diseases; diabetes; inflammatory bowel disease; and cancer [13]. According to Laplane et al., the microbiome has been defined as a part of the “tumor organismal environment” due to its capability to distally affect the TME features [9], mainly through the modulation of the host immunity within the TME. Numerous studies have further elucidated the effects of microbiome and its metabolites on additional TME characteristics, including tumor metabolism, angiogenesis and stroma remodeling [14,15,16], thus interfering with cancer progression. The existence of microbiota populations within the TME has been recognized only recently. The term intratumoral (or tumor) microbiome refers to microbial species harbored in a broad repertoire of cancer types, where they display critical roles in shaping tumor properties and defining disease outcomes [17,18,19]. The composition of microbial communities and their spatial organization within the TME have been characterized in many tumor types [17,18,20], while their crosstalk with TME components is under extensive investigation. Of note, much attention has been paid to the potential ways of harnessing the intratumoral microbiome in prognostic, diagnostic and therapeutic approaches against malignancies.

Despite the spark of knowledge in the field, the interplay between tumor microbiome and the various components of the TME has not been fully elucidated yet. Here, we overview the existing knowledge on this complex interplay, focusing mostly on the effects of intratumoral microbiome on the distinct hallmarks of the specialized tumor microenvironments, as described above [10]. Apart from this, we attempt to portray the multileveled action of the intratumoral microbiome in the TME and its concomitant impact on the dissected sub-microenvironments, which eventually drive the remodeling of the TME landscape.

## 2. Types of Specialized Microenvironments in the TME

### 2.1. Immune Microenvironment

One of the most significant, specialized microenvironment in the TME is undoubtedly the immune microenvironment (TIME). The TIME contains various types of distinct immune populations, including both cells of the innate immunity, such as macrophages, dendritic cells (DCs), neutrophils, natural killer (NK) and myeloid-derived suppressor cells (MDSC), along with cells of adaptive immunity, such as T and B cells [2]. Taking into consideration the differential representation of each of these populations in various tumor tissues, as well as their opposing functions in promoting or suppressing tumor progression [21], it becomes clear that there is high cellular and functional variation in the TIME compartment. On top of that, there is increased crosstalk between TIME-associated cells and other cellular populations of the TME, which bidirectionally affects the phenotypic characteristics of the involved cell types and eventually the tumor progression. In addition to the cellular abundance within the TIME, immune cells also secrete a great variety of factors, such as chemokines and cytokines, which orchestrate cellular communication between different cellular components of the TME and interfere with cell signaling pathways, thus affecting cancer-related processes, including cell survival, proliferation and migration [2,22]. Other components that could be also enlisted in the TIME are immunoglobulins and extracellular vesicles produced by immune cells, exerting beneficial or inhibitory effects on tumor development [2,23]. 

### 2.2. Metabolic Microenvironment

Cancer metabolism is another important element of the TME, constituting a distinct compartment of the tumor milieu. The metabolic microenvironment encompasses the metabolites produced by cancer or other cells of the TME, such as glucose, lactate, glutamine, amino acids, lipids and reactive oxygen species (ROS) [10]. Metabolism in cancer cells is severely deregulated, leading to the reprogramming of the TME via the accumulation or deprivation of several metabolic products. For instance, tumor cells display an increased glycolytic rate, generating lactate as an end-product even under normoxic conditions—a phenomenon known as the “Warburg effect” [24]. The Warburg effect entails the presence of high levels of lactate in the TME with a concomitant decrease of glucose levels, thus “starving” other cell types in the tumor milieu, especially T cells, from this nutrient. A different aspect of tumor metabolism is the dependence on glutamine, through which cancer cells meet their increased energy requirements and sustain their oxidative homeostasis, mainly by the biosynthesis of the reducing agent glutathione [25]. This is a need arising from the elevated production of ROS by the tumor cells, due to their augmented metabolic rate or the impairment of their mitochondrial genome. The abundance of ROS in the TME leads to the adaptation of cancer cells to increased ROS levels, while the increment in ROS further contributes to cancer cell proliferation, thus driving cancer progression [26]. 

In general, cancer cells are able to thrive in high-ROS TMEs, as they can manage oxidative stress better than normal cells. ROS protect and promote tumor cell survival and growth by inducing adaptive antioxidant defense mechanisms (e.g., upregulation of glutathione and superoxide dismutase aiming to neutralize excess ROS), pro-survival pathways like NF-κB and PI3K/Akt and favorable TME modifications that enhance angiogenesis [27,28]. The lack of such effects on surrounding normal cells is mainly attributed to the selective activation of the antioxidant defense mechanisms only in cancer cells, as normal cells are not continuously exposed to the same metabolic stresses as cancer cells; therefore, their baseline ROS production is lower, and the need of upregulating antioxidant defenses is minimal. In addition, normal cells possess efficient ROS-scavenging systems under homeostatic conditions that are sustained by the spatial separation and compartmentalization of ROS production within the TME [29,30,31]. Moreover, tumor cells ensure the avoidance of widespread redox system stimulation without triggering scavenging responses in the TME, by enzymatically controlling ROS production and localized consumption and by selectively inhibiting antioxidant enzymes in the surrounding normal cells to maintain a pro-tumorigenic environment [32,33].

### 2.3. Hypoxic Microenvironment

Hypoxia represents a salient hallmark of the TME that arises from the deprivation of oxygen supplies in the tumor milieu due to the rapid tumor cell proliferation. Although the elevated energy demands of the growing tumor instruct the formation of new vessels in the tumor area, the process of neovascularization is highly unsuccessful in tumors, leading to the generation of immature and “leaky” vasculature, which further supports hypoxia. In addition, the dysregulation of angiogenesis-related factors hinders the proper vessel formation to supply oxygen and nutrients to the growing tumor mass [34]. Hypoxia displays high heterogeneity within the TME, as there is usually a gradient in oxygen concentration depending on the proximity to blood vessels, which shapes the formation of hypoxic niches. Hypoxia also induces the stabilization of the transcription factor Hypoxia-Inducible Factor-1 (HIF-1) in cells, in order to activate a cell signaling cascade to counteract the low availability of oxygen and adapt their metabolic circuits [35]. On the other hand, several angiogenic factors, including the vascular endothelial growth factor-A (VEGF-A), platelet-derived growth factor-beta (PDGF-β) and angiopoietin 2, are involved in the regulation of the hypoxic microenvironment, while their expression is increased in hypoxia, mainly by HIF-1 transactivation [35]. In general, hypoxia triggers extensive reprogramming of different cell populations present in the TME, and especially of the tumor cells, increasing their stemness and aggressiveness, while it serves as a negative prognostic marker for overall survival and progression-free survival [10,36]. 

### 2.4. Acidic Microenvironment

An imminent consequence of the aberrant metabolism and the hypoxic profile of the TME is the phenomenon of tumor acidosis. The TME nurtures a slightly acidic niche, where pH values range from 6.7 to 7.1 extracellularly, while the intracellular pH values in cancer cells are slightly elevated, approximately at 7.4 [37]. This condition profoundly impacts the physiology of tumor, immune and stromal cells of the TME, endowing them with cancer-promoting characteristics and fostering tumor progression [38]. The low oxygenation in the TME along with the Warburg effect promote glycolysis in tumor cells, triggering increased lactate production, whose low pKa value contributes to TME acidification. Apart from lactate, other acidic metabolic byproducts, such as protons and CO_2_, are generated by the tumor cells, causing further exacerbation of acidosis [38]. 

Under these conditions, the regulation of tumor cell pH is largely dependent on a series of membrane transporters, in order to maintain sustainable levels for cell survival and proliferation. Lactate transporters, including monocarboxylate transporter 1 (MCT1), which mediates the import of lactate in tumor cells [39], and monocarboxylate transporter 4 (MCT4), which functions as lactate exporter [40], are instrumental to the pH regulation in the TME. In addition, several proton transporters, such as the Na^+^-H^+^ antiporter and the vacuolar-type H^+^-ATPase, contribute to the alkalization of the intracellular pH levels and the concomitant acidification of the tumor milieu [41]. On top of these mechanisms, carbonic anhydrases (CA), especially CA IX/XII, which are upregulated in solid tumors [42], act by neutralizing the diffused, extracellular CO_2_ to H^+^ and HCO_3_^−^, thus equilibrating the pH homeostasis of the tumor niche. Interestingly, the expression of lactate transporters, carbonic anhydrases and the Na^+^-H^+^ antiporter is induced by the hypoxia master regulator, HIF-1, thus underscoring the interconnectivity of the hypoxic and the acidic sub-microenvironments [43]. 

### 2.5. Mechanical Microenvironment

One indispensable part of the TME is the mechanical sub-microenvironment within the tumor stroma with all its cellular and non-cellular components. The mechanical microenvironment contains the dense ECM, which surrounds the tumor mass, and numerous cell types, including CAFs, mesenchymal stromal cells, chondrocytes and osteoblasts [44]. In comparison with the normal ECM, the tumor ECM displays high stiffness, due to increased production and deposition of ECM components, while it is heavily remodeled via several mechanisms, including the degradation of the structural networks formed by its components under the action of specialized enzymes, such as hyaluronidases, metalloproteinases (MMPs) or elastases [45]. 

Fundamental components of the ECM include structural proteins, like different types of collagen, adhesion glycoproteins (elastin, fibronectin and laminin), and various proteoglycans and glycosaminoglycans, such as hyaluronic acid. These components interconnect the two structural compartments of the ECM: the basement membrane and the interstitial matrix [44,46]. The tumor ECM further interacts with transmembrane receptors, primarily integrins, which bridge the intracellular and cytoskeletal components with ECM. Integrins can also bind cell surface adhesion molecules, such as vascular cell adhesion molecules (VCAMs) and selectins, thus mediating cell–cell adhesion and ultimately orchestrating the mechanics of the tumor tissue [47]. Additionally, intercellular adhesion can be achieved by the crosstalk of cadherins among adjacent cells. Noteworthy, in the context of the TME, there is an interplay between downstream integrin and cadherin signaling, which shapes the mechanical features of the tissue and dictates tumor motility and invasiveness [48]. From the cellular perspective, CAFs is the primary cell type in the mechanical microenvironment, displaying migratory and metabolically active phenotype by synthesizing ECM, cytokines and MMPs, which could either support or impede tumor progression [46]. They are composed of multiple distinct cell populations whose main attribute is the production of α-smooth muscle actin (α-SMA), as they display phenotypic characteristics of both fibroblasts and smooth muscle cells [45,46].

### 2.6. Innervated Microenvironment

A previously underappreciated compartment of the TME, known as the innervated microenvironment, has lately started to gain attention. An increasing number of studies highlights the presence of nerve fibers in the tumor milieu, not only in tumors of neurological origin but also in other solid tumors, such as breast and pancreatic malignancies [49,50]. The two main mechanisms of tumor innervation include neurogenesis, where neural stem cells translocate from the central nervous system to the tumor region, in order to differentiate into neurons, and axonogenesis, during which peripheral neurons are attracted and outgrow in the TME triggered by tumor-secreted molecules like neurotrophins [51]. The tumor-infiltrating neurons can be sensory, adrenergic or cholinergic. Each of the aforementioned neuron types drive distinct disease outcomes, depending on the tumor type [52], while their existence in the TME promotes neural signaling through the production of neurotransmitters and neuropeptides and their cognate receptors on tumor cells, thus leading to tumor progression [52,53]. Another feature of the innervated microenvironment involves the perineural invasion, which is defined as the entry of tumor cells into the peripheral nerves, ultimately enabling the tumor dissemination to distant regions [51]. In most tumor types, perineural invasion serves as a negative prognostic marker, associated with tumor aggressiveness and poor survival rates for cancer patients [51].

**Figure 1:**
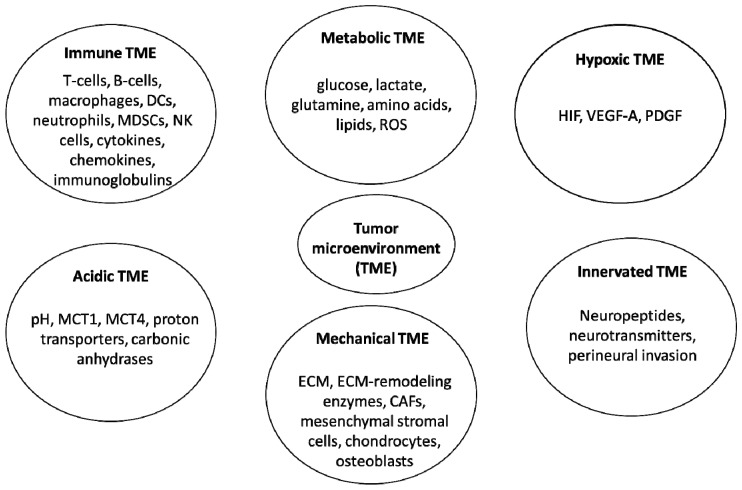
Diagrammatic representation of the six specialized sub-microenvironments found in the TME, accompanied with their main features.

An overview of the six specialized sub-microenvironments within the TME, along with their main components, is illustrated in Figure 1.

## 3. Human Microbiome: Origin, Composition, Localization and General Functions in Cancer

### 3.1. Gut Microbiome 

The gut microbiome displays high heterogeneity in terms of composition and richness between individuals, depending on the age, diet or disease state and even in different anatomic locations along the gastrointestinal tract of the same individual [13]. Despite these variations, gut microbiome primarily consists of several microorganism kingdoms, including bacteria, fungi, viruses, archaea and bacteriophages [12,13]. In the context of cancer and especially TME, there is a vast number of studies unveiling a role of gut microbiome in the regulation of distinct TME compartments.

The modulation of the host immunity by microbiota within the various TME compartments has been primarily evidenced in the case of dysbiotic gut microbiome profiles in inflammation-associated colorectal cancer (CRC) mouse models. Dysbiotic microbiome was able to promote intratumorally exhausted populations of PD-1+/Lag-3+ and PD-1+/Tim-3+ CD8+ cells, expressing low interferon-γ (IFNγ) levels, thus potentiating tumorigenesis [54]. In contrast, the presence of *Escherichia coli* strain 541-15 in the murine gut reduced the colon adenocarcinoma (CRC) incidence, as well as the tumor volume, by hindering the infiltration of tumor-associated macrophages (TAMs), regulatory T cells (Tregs) and different subtypes of MDSCs, while promoting the infiltration of type 1 helper T cells (Th1), cytotoxic T cells and type 1 innate lymphoid cells (ILC1s) in the TME [55]. In this line, the infiltration of NK cells and cytotoxic T lymphocytes in the tumor milieu was enhanced in mice whose intestines were colonized with *Helicobacter hepaticus*. Furthermore, *H. hepaticus* gut colonization induced the development of *H. hepaticus*-specific CD4+ T follicular helper cells in colorectal tumors, which drove the formation of tertiary lymphoid structures in the colonic lamina propria and controlled the tumor growth [56].

The role of metabolites produced by gut microbiota is also prominent in shaping distinct compartments of the TME, including the immune, metabolic, hypoxic, acidic, mechanical and innervated microenvironments. Notably, CD8+ T cells treated with *Megasphaera massiliensis*’ culture supernatants highly enriched in the Short-Chain Fatty Acids (SCFAs) pentanoate and butyrate, displayed increased IFN-γ and tumor necrosis factor-α (TNF-α) production, while, when compared to non-treated cytotoxic CD8+ T cells, they showed increased tumor reactivity and persistence *in vivo*, following adoptive cell therapy in mice bearing melanoma tumors [57]. Similarly, co-culture of the colon adenocarcinoma cell line HT-29 with *Fusobacterium nucleatum* strains in vitro resulted in increased secretion of formate by *F. nucleatum*, followed by augmented glutamine and glutamic acid metabolism of tumor cells. These findings, which were further corroborated in a cohort of CRC patients with high intestinal *F. nucleatum* load, highlight glutamine as the primary carbon source utilized by cancer cells, while they provide insights on the plausible effects of this gut commensal on the tumor cell metabolism in the colorectal TME [14]. The gut microbiome can also impact the extracellular matrix of the TME, as a competitive relation between gut *Bacteroides thetaiotaomicron* and the proteoglycan biglycan for the glycosaminoglycan, chondroitin sulfate, has been described in colitis-associated CRC mouse models. In this case, biglycan is glycosylated by chondroitin sulfate, whereas *B. thetaiotaomicron* is involved in the degradation of the chondroitin sulfate, thus preventing the glycosylation of biglycan. Simultaneously, chondroitin sulfate was found to propel the growth of *B. thetaiotaomicron*, ultimately interfering with CRC development [16]. 

Although a direct effect of the microbiome on the acidic TME has not been clearly elucidated; nonetheless, the presence of microbial metabolites such as the gut microbiome-derived bile acid, deoxycholate, has been described in the breast TME with the potential of contributing in lowering TME pH [58]. The gut-derived microbial metabolite trimethylamine N-oxide (TMAO) was shown to have a pro-angiogenic effect in vitro in CRC cell lines and *in vivo* in CRC tumor-bearing mice by inducing VEGF-A expression [15], thus emphasizing the effects of gut microbiome on the hypoxic TME. The role of microbiome in tumor innervation remains obscure but there is evidence of gut microbial modulation on a specific subset of enteric-associated neurons, in terms of population size and neuropeptide secretion [59], thus fueling the speculation that gut microbiome might also influence the innervated TME.

### 3.2. Skin and Oral Microbiome

Apart from gut commensals, skin commensals can also influence tumor immunity in the TME. Mice skin colonization with the engineered *Staphylococcus epidermidis* NIHLM087 strain, expressing ovalbumin antigens, could elicit antigen-specific CD8+ T cells, capable of infiltrating the B16-F0-OVA melanoma tumors and driving tumor volume reduction. Interestingly, OVA-specific CD8+ T cells primed with the *S. epidermidis*-expressed ovalbumin had a phenotype of effector or effector memory cells, compared to the ones primed with tumor-derived ovalbumin antigens in control mice, which bore predominantly central memory phenotype, thus underlining the prospect superior effects of commensals in antitumor immunity [60].

Oral microbiome is also implicated in the modulation of TME properties, as oral-resident microorganisms, including *Fusobacterium nucleatum* and *Porphyromonas gingivalis*, are capable of translocating to tumor sites and promoting tumor development, via modulation of the tumor immunity [61,62]. 

### 3.3. Blood Microbiome

An emerging concept in microbiome research supports the existence of microbiota in the blood circulation, even in healthy individuals without being associated with a clinical condition, despite having been considered a sterile environment for years. The concept of a healthy blood microbiome remains highly controversial, as in spite of numerous studies validating this concept [63], recent findings from a large-scale cohort of almost 10,000 healthy humans challenge the notion of a core microbiome associated with human blood [64]. Nonetheless, the presence of a microbiome in the blood of cancer patients has been described in many studies and attributed diagnostic and prognostic value. For instance, the detection of specific, circulating microbiome profiles in blood samples of myeloid malignancies patients was correlated with the tumor subtype, underlining their importance as a potential diagnostic tool for patient stratification [65]. Furthermore, there are indications that the blood microbiome could also serve as a prognostic biomarker in cancer patients, as its higher diversity prior to immunochemotherapy in CRC patients could predict for increased progression-free survival (PFS) and overall survival (OS) [66]. Notably, recognizing blood circulation as a potential additional microbiome reservoir in the human body could transform our understanding of the human microbiome. This insight is especially significant for the intratumoral microbiome, suggesting that blood might not merely transport microbiomes to peripheral tissues, but could also serve as a primary source.

### 3.4. Intratumoral Microbiome 

The discovery of microbiome within tumor tissues dates back to the early 20th century, when W.E. Guy reported the cultivation of viruses derived from tumors, which he further associated with tumorigenic properties [67]. However, one of the first reports concerning the antitumor action of microbiota upon administration in the tumor site was provided by William B. Coley even earlier, in 1893 [68]. These premature, contradictory studies set the scene for the characterization of the duplicitous role of the intratumoral microbiome a long time ago. However, the presence and role of intratumoral microbiome in several tumor types were only recently appreciated. 

The origin of the tumor microbiome has been controversial and might depend on the tumor type. One of the main reservoirs for intratumoral microbiome is the intestine, whose permeability can be deteriorated under certain circumstances, such as gut dysbiosis, promoting the dissemination of microbiota to distant tissues through the hematogenous route [69,70]. Another exquisite example of a distant, microbial translocation is provided by the oral-derived *Fusobacterium nucleatum*, which colonizes CRC tumors via the blood circulation [71]. Disrupted mucosal sites also favor the spread of intratumoral microbiome. In this vein, it was reported that intratumoral bacteria might migrate retrogradely from the duodenum to the pancreatic tissue through the pancreatic duct, in pancreatic ductal adenocarcinoma (PDAC) [72]. This migration is considered to be primarily reinforced by the pH gradient and to a lesser extent by the oxygen gradient between the two sites, conducting bacteria to the more neutral pH in the pancreatic tumor [73]. Another considerable source of intratumoral microbiome is the microbiome of the healthy tissue, adjacent to the tumor site, supported by the striking resemblance of their composition profile in many tumor types [17]. For instance, a study on the lung intratumoral microbiome suggested that it originates from the airways rather than the blood, due to the increased bacterial burden in the airways [74]; nonetheless, the translocation of gut commensals into lung tumor tissues via the blood circulation has also been supported in mouse models [69]. Noteworthy, the intracellular localization of bacterial species in tumor cells also contributes to their transport to metastatic niches along with the circulating cancer cells, as described in a murine breast cancer model [75]. 

The microbial communities within the tumors and those in the adjacent, normal tissue usually differ in terms of phylum, order or genus. Notably, the relative abundance of specific microbial species in a tissue can be a biomarker of a certain type of malignancy, as in the case of the taxon *Chloroplast* in renal cell carcinoma (RCC), whose relative abundance value below 0.2345% in renal tissues correlates with high sensitivity and specificity to RCC [76]. In some cases, the intratumoral microbiome signature can be correlated with the clinicopathological parameters of the tumor, as evidenced in head and neck squamous cell carcinoma (HNSCC) [77] and in esophageal carcinoma [78], or be predictive of certain subtypes of a tumor. For instance, in ESCA, an intratumoral signature of ten microbial features was predictive of the ESCA subtypes, esophageal squamous cell carcinoma (ESCC) and esophageal adenocarcinoma [78]. Furthermore, a pronounced difference in intratumoral microbiome populations at the genus level was detected between the upper and lower gastrointestinal tract tumors, distinguishing the different origin of tumors [79].

Certain intratumoral microbes might correlate with the development and progression of cancer, such as *Fusobacterium* species in gastrointestinal tumors [61]. The percentage of tumors of a specific type that are positive for intratumoral bacteria may also vary, spanning from 14.3% in melanoma to over 60% in breast and pancreatic tumors [17]. Interestingly, the intratumoral microbiome is reported to display remarkable heterogeneity even between regions of the same tumor, in colorectal cancer or adenoma [80]. In addition to this, an alteration of the intratumoral microbiome profile during the progression of adenoma to carcinoma is described in the same study [80], reflecting the heterogeneity of intratumoral microbiome in terms of the disease stage. 

Intratumoral bacteria can be found both extracellularly and intracellularly in tumor tissues [81,82], as they can invade cancer and immune cells in the TME. Gram-negative bacteria are found in the cytosol of both cancer cells and macrophages [17,83], whereas Gram-positive bacteria are primarily detected inside the macrophages and in a less extent in cancer cells [17,84]. Notably, intratumoral Gram-positive bacteria are often encountered in a cell wall-deficient state when they reside in the cancer cells [17]. Characteristically, in hepatocellular carcinoma (HCC), bacterial species were present in the cytoplasm of hepatocytes, while in peritumoral tissue, bacterial DNA was enriched in red blood cells inside the liver sinusoid. Furthermore, the intratumoral and peritumoral microbiome of HCC were more abundant in bacterial species, in comparison with their normal counterparts [85]. Tumor cell invasion by bacteria can instigate the secretion of cytokines, as in the case of *Fusobacterium* strains in PDAC, which affects the proliferative and invasive capacity of cancer cells in an autocrine or paracrine manner [86]. An interesting finding, derived by the analysis of *Fusobacterium* species in colorectal and paired metastatic liver tumors demonstrated that the presence and relative abundance of *Fusobacterium*, as well as the co-occurring dominant genera, were significantly correlated between the two sites, thus supporting the predominant intracellular localization of the tumor microbial species [87]. In addition, the composition of the intratumoral microbiome has also been implicated in the regulation of tumor metastasis by modulating the cytoskeletal scaffold of cancer cells. Moreover, it has been reported that tumor-resident bacteria promote the metastatic spread of murine breast tumor cells by reinforcing their viability through cytoskeletal reorganization after fluid shear stress in blood vessels during intravasation, which eventually enhance their colonization in distal tissues [75]. 

The above findings indicate a close association between the microbiome in primary tumor and metastatic lesions, while they suggest that the intratumoral microbiome presumably migrates along with the tumor cells to the metastatic sites.

#### 3.4.1. Intratumoral Fungi and Viruses

Similarly, the presence of intratumoral fungi species in the TME, although not as frequently observed as bacteria, is predominantly detected within the cancer or immune cells, depending on the tumor type, whereas their extracellular localization is less detected [20]. In general, the intratumoral mycobiome species reflect the fungal composition of the specific tissue but differential abundances are described between normal and cancer tissue or between different cancer stages [20]. Surprisingly, and in sharp contrast with the competitive relationship between fungal and bacterial populations that has been previously described in the gut, bacteria and fungi seem to co-exist in the TME, displaying positive correlations in terms of diversity and abundance, thus indicating that the TME probably constitutes a non-competitive milieu for microbial species, termed as a “permissive” phenotype [20,88]. For instance, significant associations between the tumor-associated fungal genera *Candida* and *Saccharomyces* and distinct bacterial populations were described in gastrointestinal tumors, shedding light on the possible crosstalk between the different microbiome components in the context of the TME [89].

A growing body of evidence also suggests the existence of several viral strains in the TME of various tumor types, including poxviruses and papilloma viruses in triple-negative breast cancer (TNBC) [90], *Ortevirales* in HNSCC [91], HSV in soft tissue sarcomas [92] and CMV in CRC tissues [93]. However, more comprehensive studies deciphering the intratumoral viral landscape in different malignancies are still lacking. In addition, low intratumoral abundance of archaea is detected in the TME of soft tissue sarcomas [92], but more extensive investigation on intratumoral archaea is required. The potential presence of intratumoral protozoa has also not been explored, despite the efforts in harnessing protozoan parasites in adjuvant cancer therapy [94].

#### 3.4.2. Determinants of Intratumoral Microbiome Abundance

Numerous factors can dictate or influence the qualitative or quantitative profile of intratumoral microbial communities. For example, it has been reported that race might be an important factor in the composition of the tumor microbiome, as significant correlations between race and intratumoral microbial abundances have been recognized in a number of malignancies [95]. Neoadjuvant chemotherapy has been also shown to decrease bacterial diversity and alter bacterial abundances in the TME of breast cancer [96]. Apart from this, patient the body mass index has also been implicated in differences in intratumoral bacterial species, in the context of breast cancer [96]. Importantly, intratumoral microbiome communities can also be associated with the mutational landscape of the cancer cells, like the associations established between *Bacteroides* and *KRAS* mutations or Proteobacteria and microsatellite instability in CRC tissues [80]. In the case of the basal-like subtype of pancreatic cancer, a distinct microbiome signature of the genera *Acinetobacter*, *Pseudomonas* and *Sphingopyxis*, was positively associated with several cancer cell signaling pathways, such as Kras, MAPK and epithelial-to-mesenchymal transition, as well as with pathways controlling the response to bacterial components, such as lipopolysaccharide, thus highlighting the effects of intratumoral microbiome on both tumor cells and host immunity [97]. Noteworthy, a pan-cancer intratumoral microbiome study suggested a microbiome-based scoring system, evaluating 64 intratumoral microbial taxa to divide the patients in distinct subgroups, significantly associated with the host genomic landscape, the immune infiltration and the disease prognosis, therefore underscoring the impact of intratumoral microbiome on factors affecting the tumor progression [98]. 

#### 3.4.3. Impact of Intratumoral Microbiome-Produced Metabolites on Cancer Properties 

The intratumoral microbiome, as described above, is possible to indirectly exert its effects on TME remodeling through its metabolic products. In this context many bacterial species, which inhabit human tumors, carry out lactate production as a fermentation end product, including the dominant phyla of Bacteroidetes, Firmicutes and Proteobacteria [17,99,100]. Indeed, lactate accumulation has been observed in many cancer types [101,102], contributing to acidification of the TME due to its low dissociation constant pKa 3.85. The production of SCFAs is another metabolic feature of specific bacterial genera. SCFAs are organic acids comprising acetate, propionate and butyrate, and they are fermentation products of carbohydrates [103]. Butyrate has been reported to exert an anti-proliferative effect in cancer cells mainly by acting as a histone deacetylase (HDAC) inhibitor [104]. Similarly, microbiome-produced propionate contributes to tumor cell growth attenuation in glioblastoma multiforme (GBM) mouse xenografts and this effect is mediated by the peroxisome proliferator-activated receptor type-γ (PPAR-γ) [105]. The majority of bacteria found in human tumors are capable of synthesizing SCFAs, such as species belonging to phyla Bacteroidetes and Firmicutes, as well as the anaerobic genera *Fusobacterium* and *Bifidobacterium* [17,103,106]. For example, the strain *Megasphaera* sp. XA511, a member of the bacterial genus *Megasphaera* abundant in PDAC tissues, was verified to produce butyric acid [100], while increased butyrate accumulation has been also detected in the TME of oral squamous cell carcinoma (OSCC) [101]. Butyrate is further produced by the bacterial genera *Finegoldia* and *Nocardioides*, found also in PDAC tissues isolated by long-term PDAC survivors [107]. In this line, intratumoral *Propionibacterium acnes*, detected in epithelial ovarian cancer tumors [108], is a major producer of propionate [109], which is also abundant in CRC tissues [102]. Intratumoral bacterial species, including *Lactobacillus reuteri* and *Bacteroides thetaiotaomicron* [100,110], are also endowed to synthesize tryptophan metabolites, such as indole-3-acetic acid (3-IAA) and indole-3-aldehyde (I3A), whose presence in the TME could influence immune features and metabolic signaling pathways [110,111], as explained below. Similarly, polyamines metabolites produced by the *Enterococcus faecalis* and *Escherichia coli* bacterial species can be found in the TME, thus supporting the hypothesis that they could originate from intratumoral microbiome [112]. 

In the following sections, we attempt to illustrate in detail the direct and indirect impacts of the intratumoral microbiome and its products on the remodeling of the TME landscape through a complex interplay with the distinct and specialized TME microenvironments, as analyzed above. 

## 4. Crosstalk between the Intratumoral Microbiome and the Tumor Immune Microenvironment (TIME) 

### 4.1. Microbiome as a Driver of TME Immunostimulation 

Recent evidence derived by a murine breast tumor model suggested that immunodeficient mice bore more extracellular tumor bacteria than immunocompetent mice, thus underlining the complex interplay between the tumor microbiome and the systemic immune system [75]. Accordingly, the presence of intratumoral bacteria has also been detected in tumors located in immune-privileged sites, such as ovarian cancer and GBM [17]. In this context, the impact of the intratumoral microbiome on the tumor-suppressive properties mediated by certain immune cell populations within the TME has been well described at multiple levels, including the infiltration, activities and phenotypic alterations of the immune cells that crosstalk with the tumor cells (Supplementary Table S1A).

#### 4.1.1. TME Infiltration by Immune Cells

In CRC patient tissues, a positive correlation was observed between microbial enrichment in the tumor core and CD3+ T cell infiltration (TILs). Specifically, nine bacterial species, including members of the *Blautia*, *Faecalibacterium* and *Faecalitea* genera, were found to be highly abundant in the tumor core of patients with elevated CD3+ T cell infiltration [113]. Similarly, the relative abundance of the viral microbiome in soft tissue sarcoma tumors is associated with increased NK cell infiltration within the TME, which further entails improved overall patient survival [92]. The intratumoral microbiome of PDAC patients with long-term survival is also characterized by the abundance of the bacterial taxa *Sachharopolyspora*, *Pseudoxanthomonas* and *Streptomyces*, which correlate with increased CD8+ T cell infiltration and greater production of the apoptosis effector molecule granzyme B [19]. In this context, findings derived by an analysis of a melanoma patient cohort from The Cancer Genome Atlas (TCGA) revealed that sixteen intratumoral bacterial genera, including the *Lachnoclostridium* genus, were positively associated with CD8+ T cell infiltration, attributed to increased expression of the chemokines CXCL9, CXCL10 and CCL5, thus resulting in better patient overall survival rates [114]. 

Likewise, the prevalence of *Fusobacterium nucleatum* in human tumor tissues of OSCC was inversely correlated with the infiltration of B cells, CD4+ T helper cells and type 2 macrophages (M2), sculpting a less immunosuppressive TME and favoring the disease prognosis [83]. In a similar context, the intratumoral abundance of *Streptococcus* was associated with higher infiltration of CD8+ and granzyme B+ T cells in the TME of ESCC patients, while it was found enriched in tumor tissues derived by patients responding to neoadjuvant chemoimmunotherapy, thus suggesting a putative predictive value in disease-free survival [115]. Furthermore, in a clinical study of patients with advanced refractory solid tumors, a single intratumoral injection of non-toxic *Clostridium novyi* spores promoted immune cell infiltrates, comprising effector CD8+ T cells, Tregs and MDSCs, while it manifested with tumor cell rupture and other clinical indications [116]. 

The above findings suggest that intratumoral bacteria might render the tumors more immunologically “hot”, thus opening windows for novel combinational therapeutic approaches with immunotherapy. In this line, the intratumoral injection of the attenuated rhino/poliovirus chimera, PVSRIRO, in combination with intraperitoneal anti-PD-L1 administration, in mice bearing B16.F10 melanoma tumors led to TME enrichment not only in CD4+ T cells, but also in cytotoxic populations of NK and CD8+ T cells that are characterized by increased granzyme B and IFN-γ secretion rates. Those antitumor effects were mainly mediated by activation of innate immunity responses through induction of interferons type I and III, following the viral infection [117]. Similarly, the intratumoral administration of the protozoan parasite *Neospora caninum* in mice bearing melanoma B16.F10 tumors led to augmented CD8+ T cell and CD68+ macrophage infiltration in the TME, accompanied with increased levels of Th1 cytokines in the tumor milieu, such as IFNγ, TNFα, IL-2, IL-10 and IL-12, therefore resulting in a reduction in tumor volume [94]. 

#### 4.1.2. Antitumor Immune Cell Activities

One of the most well-studied cancer models that crosslink the intratumoral microbiome composition with efficient antitumor immune cell responses is melanoma. Several bacterial species have been identified in the TMEs of melanoma patients, while bacterial peptides from these species were found to be presented on either HLA-I or HLA-II molecules, by both tumor and antigen-presenting cells. These findings were further corroborated in vitro, in various melanoma cell lines stimulated with bacteria-derived peptides and co-cultured with CD8+ Τ cells. Following cancer cell stimulation, TILs responded with augmented secretion of IFN-γ, thus implying increased reactivity [84]. A similar study in GBM tumor tissues revealed that bacterial peptides, derived mainly by the GBM-abundant phyla Firmicutes and Proteobacteria, were presented as complex with HLA-class II antigens expressed on tumor cells, thus stimulating multiple TIL-derived CD4+ T cell clones for secretion of proinflammatory cytokines and chemokines [118]. Moreover, in mouse melanoma tumor models, the translocation of the gut commensal *Lactobacillus reuteri* in the melanoma sites or the direct intratumoral injection of *L. reuteri*, enhanced the type 1 cytotoxic T cell (Tc1) phenotype within the TME and resulted in inhibition of tumor growth. The underlying mechanism was dependent by *L. reuteri*-derived I3As, as I3A augmented the aryl hydrocarbon (AhR) receptor signaling in CD8+ T cells, which in turn stimulated the secretion of IFNγ, thus driving the antitumor immunity [110]. 

In the same context, the intratumoral accumulation of the *Bifidobacterium* genus bacteria members in mice bearing other cancer types, including MC38 CRC tumors, triggered activation of the STING-mediated type I IFN signaling in dendritic cells of the TME, which in turn accelerated the efficacy of the anti-CD47 immunotherapy in a T cell-dependent manner [119]. Similarly, the presence of microbiome in mice with lymphoma skews the TME towards stimulatory monocytes and dendritic cells, whereas it limits the number of suppressive macrophages by generating products, such as the STING agonist cdAMP, which regulate the axis type I IFN-NK-dendritic cells and sustain a proinflammatory TME to promote antitumor immunity [120]. Moreover, mice bearing HCC tumors showed diminished expression of IL-17A by hepatic type 3 innate lymphoid cells (ILC3s) when they were administered with fecal microbiome from healthy mice or *Lactobacillus reuteri*, which further implicated delayed tumor growth. In essence, the inhibition of IL-17A production in hepatic ILC3s was found to be mediated by SCFAs, synthesized in the colon and metabolized by gut bacterial enzymes, such as *L. reuteri* [121], underscoring the impact of microbiome metabolites on the tumor immune niche. On a different note, the presence of a distinct tumor mycobiome profile in PDAC mouse models was necessary to drive tumorigenesis, with the genus *Malassezia* having a prominent role in the process, via the activation of the mannose-binding lectin/C3 complement cascade signaling pathway [122].

The impact of the intratumoral microbiome on the tumor suppressive function of immune cells present in the TME is further illustrated by several studies with intratumoral administration of engineered bacteria. For instance, a genetically attenuated *Salmonella* strain (VNP20009) was harnessed as a “vehicle” for tumor antigens in the melanoma TME periphery, via coating with positively charged nanoparticles. A wide range of well-known tumor-mutated proteins, including four tumor neoantigens, Ddx27, Cad, Aldh18a1 and Glud1, were able to be adsorbed on the surface of antigen-capturing bacteria, eliciting antitumor responses by activating dendritic cells in the margins of the TME, which in turn migrated to the lymph nodes for T cell priming. Subsequently, the intratumoral injection of the antigen-capturing bacteria in combination with radiotherapy enhanced the antigen-specific T cell population within the melanoma TME [123]. Similarly, an engineered strain of *Escherichia coli* Nissle 1917 conjugated with the AS1411 aptamer, which refers to a synthetic oligonucleotide endowed with the capacity to bind nucleolin targets, displayed increased localization in the TME of mice bearing 4T1 tumors, after intravenous injection, which in turn led to elevated TME infiltration by CD4+ T cells and augmented production of IFN-γ and TNF-α [124].

Further examples of TME immunostimulation mediated by engineered intratumoral bacteria are derived from the study of Wang et al., who modified an *Escherichia coli* strain to express melanin and conjugated it with anti-PD1 molecules. Upon intratumoral injection of this engineered strain in a 4T1 breast cancer mouse model, in combination with laser irradiation, the infiltration of CD45+ leukocytes along with the expression of MHC-II and CD86 on dendritic cells, as well as the intratumoral IFNγ+/CD8+ T cell population were potentiated. On the contrary, immunosuppressive populations including Tregs were significantly diminished in the TME, thus promoting a further reduction in tumor volume [125]. Similarly, the intratumoral or intravenous injection of the non-pathogenic *Escherichia coli* strain MG1655, engineered to produce TNFα, in mice bearing renal or colorectal carcinomas, respectively, altered the cytokine profile in the TME to a proinflammatory state with increased expression of IFNγ and IL-12 that ultimately led to tumor regression [126]. Moreover, intratumoral administration of the engineered *E. coli* strain Nissle 1917 expressing the activating form of the human chemokine CXCL16, hCXCL16^K42A^, into subcutaneous murine tumors of the A20 B cell lymphoma model resulted in increased proliferation of effector CD4+ and CD8+ T cells within the TME. The latter was evidenced by the elevated secretion of granzyme B and IFNγ by CD4+ and CD8+ T cells, respectively, in in vitro assays upon cell restimulation with the engineered strain. In the same setting, the addition of the chemokine CCL20 to the bacterial payload also enhanced the infiltration of dendritic cells into the TME, thus resulting in a further tumor volume reduction [127].

#### 4.1.3. Phenotypic Alterations of Immune Cells

In mice bearing melanoma tumors, certain gut microbial species have been reported to translocate to the tumor site upon immune checkpoint therapy, where they enhance the expression of the MHC-class II antigens and the CD40, CD80 and CD86 co-stimulatory receptor molecules on the surface of dendritic cells. Moreover, dendritic cells pulsed with the translocated *Enterococcus faecalis*, *Lactobacillus johnsonii* and *Escherichia coli* species promoted a significant increase in IFN-γ secretion by CD8+ cells, derived by OT-I transgenic mice [128]. Intratumoral bacterial phyla, including Firmicutes and Bacteroidetes, detected in CRC patient tissues, were negatively correlated with the expression of IL-17a and the chemokine CCL20, which is known to bind the receptor CCR6 on Th17 cells. These findings suggest a negative association between the aforementioned bacterial phyla with the Th17 immune cell phenotype in CRC [129]. In addition, the population of type 1 macrophages (M1) present in the TME of ovarian cancer patients was positively associated with the protective, intratumoral microbiome species *Achromobacter deleyi* and *Microcella alkaliphila*, while it was negatively associated with the risk species *Devosia* sp. LEGU1, *Ancylobacter pratisalsi* and *Acinetobacter seifertii*. In this context, in vitro treatment of M1 macrophages with *Acinetobacter seifertii* impeded the migratory potential of these cells, thus indicating an underlying mechanism explaining the inverse correlation of these intratumoral bacterial species with the M1 phenotype [130]. 

Moreover, in tumor tissues derived by long-term PDAC survivors, the intratumoral loads of the genera *Bacillus* and *Paenibacillus* were significantly elevated compared to those from short-term PDAC survivors [19,131]. The above genera synthesize the enzyme CutC, which contributes to the synthesis of the bacterial metabolite TMAO. The administration of TMAO in an orthotopic PDAC mouse model resulted in skewing several immune populations within the TME, including TAMs, MDSCs, dendritic cells, and CD4+ and CD8+ T cells, towards more immunostimulatory phenotypes. This switch was achieved via extensive transcriptional remodeling, that ultimately induced the expression of activation markers or hindered the expression of immunosuppressive markers. Specifically, the effect of TMAO on TAM transcriptional reprogramming towards an immunostimulatory phenotype and an antitumor function was shown to be Type-I IFN dependent. A similar proinflammatory impact of TMAO was further detected on human macrophages under conditions mimicking the PDAC milieu, thus providing a causal link between the intratumoral abundance of TMAO-producing genera and the overall survival in PDAC [131].

### 4.2. Microbiome as a Driver of TME Immunosuppression

Apart from the immunostimulatory role of the intratumoral microbiome within the TME, a parallel and sometimes overlapping immunosuppressive impact of certain microbiome species has also been reported in the TME at multiple levels, as analyzed below.

#### 4.2.1. TME Infiltration by Immune Cells

A microbiome analysis of cancer tissue biopsies derived by the SHIVA clinical trial [132] revealed that reduced intratumoral bacterial richness was significantly associated with increased number of TILs; nonetheless, the limited bacterial richness was also associated with reduced overall survival (OS) and progression-free survival (PFS) [133], presumably due to the infiltration of immunosuppressive lymphocytic populations. 

Colon cancer is one of the most studied cancer types regarding the interactions developed between the intratumoral microbiome and the infiltrating immunosuppressing cell populations. Utilizing the ImmuCellAI method on data extrapolated by the TCGA CRC patient cohort, Liu et al. associated a cluster of tumors with low immune cell infiltration with the abundance of pathogenic bacterial genera, such as *Parvimonas*, *Alistipes*, *Oscillibacter* and *Tyzzerella*, while beneficial bacteria, such as *Blautia* and *Akkermansia*, were diminished. Specifically, bacteria of the *Alistipes* genus, which were highly enriched in low-infiltrated tumors, were positively correlated with CD8+ T naïve and memory cells, whereas they were negatively correlated with macrophages, NK and MAIT (Mucosal-associated Invariant T cell) cells. Other bacteria reported to be involved in CRC pathogenesis, such as *Parvimonas* and *Bilophila*, were also negatively associated with NK and MAIT cells in the TME [134], while the high load of *Fusobacterium nucleatum* in CRC tumor sites was further inversely correlated with the CD3+ T cell infiltration, thus limiting the potential of the adaptive immunity in tumor eradication [135]. In parallel, the intratumoral abundance of *Fusobacterium* species in CRC patients was positively correlated with TME infiltration by immunosuppressive innate cell populations, including TAMs, MDSCs and tumor-promoting dendritic cells. Interestingly, the same infiltration pattern of MDSCs was also observed in colorectal tumors grown in Apc^Min/+^ mice and enriched, after mouse feeding, in *Fusobacterium* species [61]. These findings indicate common effects of *Fusobacterium* on tumor immunity in human CRC tissues and CRC mouse models. Similarly, the accumulation of *Fusobacterium nucleatum* in tumor samples derived by CRC patients with high microsatellite instability (MSI) was positively associated with TME enrichment in a CD163+ macrophage population, which corresponds to the suppressive M2-polarized phenotype, whereas it was negatively correlated with FoxP3+ T cell density throughout the tumor core [136]. Those immune populations establish a pro-tumoral immune microenvironment and might interpret the increased tumor growth and invasion in CRC patients with high MSI status and increased *F. nucleatum* tumor colonization [136]. 

In a different setting, the proinflammatory role of CRC-associated bacteria in potentiating TME infiltration by B cells, through an IL-17-dependent fashion, was outlined in a mouse model of colitis-induced CRC, hence supporting tumor growth and progression. On the other hand, intratumoral polymorphonuclear neutrophils were able to suppress the bacterial outgrowth within the TME and reverse the aforementioned effect [137]. Both alpha- and beta-diversity (referring to the species richness index within a community and the species differentiation index between communities, respectively) of the intratumoral microbiome in human CRC samples were significantly associated with CD8+ T cell counts in tumor tissues [138]. For instance, the operational taxonomic unit (OTU) OTU_104, which belongs to the order Clostridiales, was inversely correlated with the population of CD8+ T cells in the tumor, while its abundance was associated with poor disease-free survival, which might be linked to the decreased CD8+ T cell infiltration [138]. Moreover, the oral administration of *Porphyromonas gingivalis* in CRC xenograft mouse models enriched the *P. gingivalis* load in the tumor tissue, while it enhanced the recruitment of CD11b+ myeloid cells, macrophages and dendritic cells in the TME, in a NLRP3 inflammasome-dependent manner [139]. Consistent with the above findings were the increased CD11b+ myeloid cell infiltration in the TME that was monitored in CRC patient tissues with higher intratumoral levels of *P. gingivalis*. Moreover, the elevated levels of the proinflammatory cytokines, TNFα, IL-6 and IL-1β in the tumor milieu of *P. gingivalis*-gavaged mice might further contribute to *P. gingivalis*-mediated CRC progression [139]. 

TME immunosuppression triggered by the intratumoral microbiome composition has been further evidenced in pancreatic carcinomas. Concomitant to CRC, enrichment of the oral cavity-resident *Porphyromonas gingivalis* was also observed in murine pancreatic cancer tissues, after oral gavage with the aforementioned bacterial species, while their intratumoral accumulation was associated with accelerated tumor progression. Suggested underlying mechanisms of tumor progression were (1) the limited infiltration of CD8+ T cells; (2) the enhanced infiltration of neutrophils in the TME, as *P. gingivalis* upregulates the expression of chemokines, such as CXCL1, CXCL2 and CXCR2, well known as neutrophil chemoattractants; and (3) the *P. gingivalis*-mediated modulation of the infiltrated neutrophil function, through induction of the production of neutrophil-derived elastase, a fundamental component of neutrophil extracellular traps (NETs) that exacerbate tumor growth [62]. The results of the study are of particular interest, considering the high abundance of *P. gingivalis* in human pancreatic adenocarcinoma tissues compared to their healthy counterparts [62]. The intratumoral strain *Acidovorax ebreus* TPSY was further associated with metastasis and high tumor grade in PDAC tissues, while the abundance of this bacterial strain was correlated with reduced infiltration of CD8+ T cells, activated memory T cells and M2 macrophage accumulation within the TME, thus implying immune suppression [140]. 

In other cancer types, the bacterial dysbiosis in patients bearing tumors of different subtypes of papillary thyroid carcinoma (PTC) has been also associated with diminished infiltration of effector immune cells in the TME, as a significant correlation was observed only for resting CD4+ memory T cells in follicular variant PTC, the one out of three PTC subtypes studied, whereas the normal tissue abounded with immune cells [141]. Moreover, a cohort study in melanoma tissues revealed negative associations between intratumoral members of the genera *Algibacter* and *Epilithonimonas* and CD8+ T cell infiltration, while the *Algibacter* genus was also negatively correlated with overexpression of the chemokines CXCL9, CXCL10 and CCL5 [114]. In this context, analysis of prostate adenocarcinoma data retrieved from TCGA showed that most microbes were more prevalent in the tumor tissue, in comparison with the adjacent normal tissue. Moreover, the intratumoral microbial abundance was significantly correlated with Tregs infiltration, while correlations established between the microbiome and TME infiltration by NK, M1 and M2 macrophages did not prove any statistical significance, thus suggesting that the intratumoral microbiome skews the prostate TME to an immunosuppressive state [142]. Distinct intratumoral microbiome composition was also recognized in cohorts of lung adenocarcinoma (LUAD) or lung squamous cell carcinoma (LUSC) patients of different age and gender. The high load of *Pseudomonas putida* strain KT2440, characteristic for the younger male LUSC patients, was associated with lower infiltration of naïve B cells and activated dendritic cells, whereas the low abundance of *Rothia dentocariosa* ATCC 17931, observed almost in all male groups and older LUSC patients of both genders, was correlated with lower infiltration levels of naïve B cells, resting mast cells, and M1 and M2 macrophages. Additionally, the low intratumoral levels of *Thermostaphylospora chromogena*, which display the same distribution pattern in LUSC patient cohorts as *Rothia dentocariosa* ATCC 17931, are further correlated with decreased infiltration of naïve B cells, resting CD4+ T and NK cells, as well as activated mast cells, reflecting the impact of different intratumoral microbiome signatures on TME immunomodulation [143]. 

On the other hand, the presence of *Actinobacter* genus members in high-risk breast cancer patient tissues was positively associated with intratumoral CD8+ T cell accumulation, despite its concomitant positive association with lymph node-positive status and metastasis. Contrarily, associations between the genus *Methylibium* and multiple immune pathways were established only in healthy breast tissues, whereas it was negatively correlated with T cell infiltration in tumor samples [144]. Moreover, mice bearing mammary tumors and fed a high-fat diet presented a higher load of intratumoral Gram-positive and Gram-negative bacterial species, along with elevated levels of TAMs. Notably, in human primary breast cancer tissues, the presence of intratumoral Gram-positive bacteria was positively correlated with TME infiltration of CD45+ leukocytes [145]. In gastric cancer, the intratumoral localization of *Stenotrophomonas*, *Acinetobacter*, *Gemella*, *Neisseria*, *Aquabacterium*, *Haemophilus*, *Novosphingobium*, *Streptococcus*, *Massilia*, *Gemmiger*, *Chryseobacterium* and *Brevundimonas* was positively correlated with high abundance of the immunosuppressive BDCA2+ plasmacytoid dendritic cell population within the gastric TME. In addition, other intratumorally found genera, such as those of *Streptococcus*, *Massilia* and *Fusobacterium*, as well as the less abundant *Oribacterium*, *Campylobacter*, *Selenomonas*, *Dialister* and *Photobacterium* genera, were correlated with TME infiltration of Foxp3+ Tregs, therefore contributing to an immunosuppressive gastric TME [146]. Furthermore, the intratumoral microbiome diversity in human ESCC tumors was inversely correlated with the accumulation of NK cells in the tumor milieu and associated with lower overall patient survival, as well [147]. 

#### 4.2.2. Tumor-Promoting Function of Immune Cells

In patients with OSCC or CRC, intratumoral bacteria display a heterogeneous distribution within the tumor, being enriched in immunosuppressive niches of the TME that contain numerous CD66b+ myeloid cells which express high levels of arginase 1 and CTLA-4. In both OSCC and CRC bacteria-positive microniches, the expression of T cell surface markers, such as CD3, CD8, CD4, CD27 and CD44, was also severely compromised, while the bacterial load in the OSCC TME microniches was further associated with PD-1 overexpression in cancer cells [18]. Similarly, the richness of intratumoral bacterial species and the relative abundance of *Lactobacillus* species as well as the intratumoral load of *Fusobacterium nucleatum* in ESCC patient tissues were positively correlated with enhanced accumulation of PD-L1+ cancer cells and PD-L1+ TAMs [147], or inversely associated with the peritumoral lymphocytic reaction [148], respectively, thus indicating features of an immunosuppressive TME.

Single-cell analysis of host–microbiome interactions in PDAC patient tissues revealed that pancreatic tumor cells harbor a vast number of different bacterial species. T cells present in PDAC TMEs enriched with bacteria were more prone to natural killer T (NKT) cells or effector phenotypes rather than to regulatory phenotypes. However, these T cells had a transcriptional profile resembling an infection status, rather than tumor stimulation, thus providing a possible explanation of why immunotherapy fails in pancreatic tumors [149]. Accordingly, the TMEs in PDAC mouse models were highly abundant in fungi species, especially those from the *Malassezia* genus, and their presence was correlated with increased tumor secreted IL-33 levels, which in turn activated type 2 innate lymphoid cells (ILC2). The elimination of intratumoral mycobiome via anti-fungal treatment resulted in limited tumor infiltration of type 2 immune cells, such as ILC2 and Th2, reduced tumor volume and improved survival rates [150]. Furthermore, metastatic PDAC tissues had been also found enriched in additional microbial species, including *Mycoplasma hyopneumoniae* and *Citrobacter freundii*, that were correlated with immune suppression pathways, while the latter strain was also positively correlated with proinflammatory immune cascades [140]. In a different setting, mice deficient in the pattern recognition receptor Dectin-3 showed a marked increase in *Candida albicans* in their colonic tissues, which induced glycolysis and IL-7 synthesis in macrophages. Subsequently, IL-7 drove the IL-22 production in ILC3s, which was primarily responsible for the *C. albicans*-dependent colitis-associated colon cancer progression [151]. 

#### 4.2.3. Phenotypic Alterations of Immune Cells

Direct evidence for the translocation of the gut microbiome to the tumor tissue, as well as on its abundance comparing with the healthy tissue, was provided by studies using various PDAC mouse models. In fact, a role in tumor progression was attributed to microbial communities, as their ablation via antibiotic administration drove a shift from immunosuppressive M2 TAMs to M1 TAMs in the pancreatic TME that concomitantly potentiated the intratumoral populations of Th1-polarized CD4+ and the cytotoxic CD8+ T cells, in a Toll-like receptor (TLR)-dependent manner [152]. In this line, further *in vivo* studies revealed that the administration of the indole-producing bacterial species, *Lactobacillus murinus* and *Lactobacillus reuteri*, in mice bearing PDAC tumors led to tumor progression by reducing the population of CD8+ T cells while increasing the number of MDSCs in the TME. These effects were attributed to indole synthesis by the aforementioned bacterial species as they were not observed after transplantation of non-indole-producing Lactobacillus species, such as *L. johnsonii* and *L. intestinalis*. Indole action on tumor progression was further suggested to be mediated by the AhR on TAMs, which accounts for the immunosuppressive phenotype of TAMs and its expression is associated with poor clinical outcomes for PDAC patients [153].

Concomitantly, in an inflammation-induced CRC mouse model, a distinct gut microbiome profile signature was associated with increased tumor burden due to the shrinkage of the intratumoral CD8+ cell population in combination with elevated levels of exhaustion surface markers and the reduction in IFN-γ production [54]. Moreover, the enrichment of the intratumoral *Methylobacterium* species in gastric cancer patients was also negatively correlated with the presence of CD8+ and CD103+ tissue-resident memory cells in the gastric TME, corresponding to poor disease prognosis [154]. In a different context, the lung intratumoral bacterial load promotes the expansion and accumulation of a distinct RORγt+, IL-17A+ γδ T cell population in the lung TME of a mouse model of human LUAD, which further produces tumor cell proliferation mediators, such as IL-22, that drive neutrophil infiltration and potentiate tumor growth [155]. Interestingly, the presence of a similar γδ T cell profile in LUAD patients’ tissues was correlated with lower survival rates, thus reflecting the clinical relevance of the lung intratumoral microbiome in reshaping the immune microenvironment within the lung TME [155].

Despite the increasing number of studies describing positive or negative correlations between the intratumoral microbiome and immune populations, activation markers on immune cells, cytokines or chemokines, there is a lack of mechanistic studies elucidating the underlying mechanism by which intratumoral microbiota modulate those immune features. There is an imperative need for more investigation to delineate those mechanistic links, to provide a better understanding of the role of intratumoral microbiome in promoting or suppressing immune activities in the TME. In this way, the dual role of certain microbes in conferring immunostimulation or immunosuppression in the same tumor type, as in the case of the *Malassezia* genus in PDAC [122,150], could be interpreted. On top of that, the comprehension of the ways of action of intratumoral microbiota in the immune TME would offer the opportunity for therapeutic targeting of specific microorganisms or other TME components modulated by them to provide a therapeutic benefit to cancer patients. 

On a different note, there is also a need for deeper understanding of the potential role of the intratumoral microbiome in modulating the effects of immunotherapy. Given the impact of the intratumoral microbiome on the immune compartment of the TME and the well-established connection between gut microbiome and immunotherapy outcomes [156], it is intriguing to wonder whether the intratumoral microbiome could also affect immunotherapy efficacy. 

**Figure 2:**
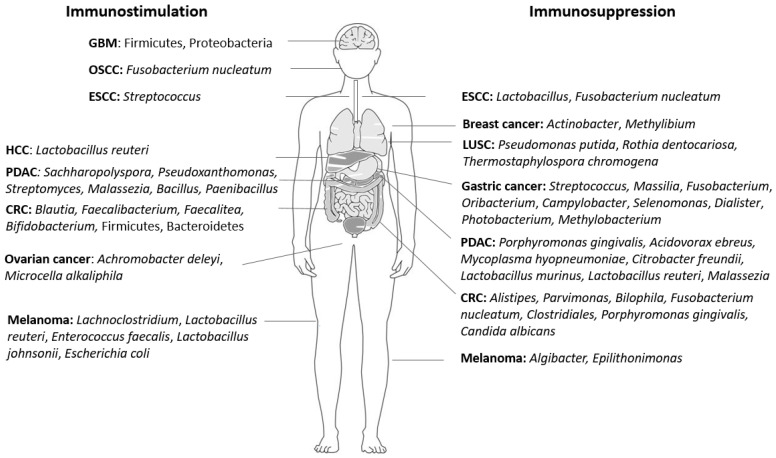
Graphic illustration portraying intratumoral phyla, genera or species, promoting either immunostimulation (left side) or immunosuppression (right side) in different tumor types. More details regarding their specific impact on the immune TME found in the text.

Figure 2 summarizes the aforementioned intratumoral microorganisms, modulating the immune compartment of the TME in different tumor types, with opposing functions.

## 5. Crosstalk between the Intratumoral Microbiome and the Acidic TME 

The pH of the TME is undoubtedly a factor that dictates the microbial composition of tumors. pH dramatically influences the viability of lactate-producing and lactate-utilizing bacteria, as a shift from pH 6.5 to 5.5 favors lactate-producing bacteria and simultaneously limits the population of lactate-utilizing bacteria [157]. At pH 6.5, which closely resembles the conditions in the acidic TME, the phyla Bacteroidetes and Firmicutes thrive in fermentation-based experiments and indeed, the predominance of these phyla is observed in several tumor types [157]. Importantly, the microbiome bears the capability of adaptation to the acidic TME by different strategies. For example, gut microbiota in CRC downregulate the expression of the Na^+^-H^+^ antiporter on their cell membranes or they limit the membrane permeability to extracellular protons by the deposition of unsaturated fatty acids [158]. It is possible that such strategies are adopted by the intratumoral microbiome to resist to the acidic microenvironment of solid tumors. Intratumoral microbiome can also directly affect the pH of the TME. 

The intratumoral load of *Fusobacterium nucleatum* in OSCC tissues triggers the acidification of the TME by enhancing the GLUT1 expression on the surface of OSCC cells, promoting glycolysis and the consequent increased secretion of lactate to the TME [159]. The *F. nucleatum*-induced lactate deposition in the TME also favors the M2-like TAM population in the tumor milieu, supporting tumor invasion [159]. As mentioned previously, intratumoral bacterial species are capable of synthesizing certain metabolic products, which have been identified in the tumor microenvironment, such as SCFAs, lactate and polyamines [101,160]. SCFAs and lactate accumulation in the TME could contribute to the decrease of the extracellular pH due to the low pKa values of these metabolites, consequently shaping an acidic TME. On the other hand, polyamines, such as putrescine, have been attributed with a role in buffering the intracellular pH of myeloid lineage cells within the acidic TME of glioblastoma tumors to facilitate their survival and functional capacity [161]. Therefore, the production of polyamines by intratumoral microbial species could lead to their uptake by tumor or immune cells within the TME, endowing them with the capacity to persist in the acidic tumor milieu. 

The effects of the secondary metabolites of microbiome on tumor cells can be also modulated by the acidic tumor pH, as it has been shown that the *Propionibacterium freudenreichii*-produced SCFAs, acetate and propionate, trigger either apoptotic or necrotic cell death in colon adenocarcinoma cells in vitro, at pH values 7.5 or 5.5, respectively [162]. Metabolites derived from the intratumoral microbiome could also play a role in the adjustment of the pH of the TME. For instance, it has been described that treatment of breast cancer cell lines with butyrate led to increased expression of the MCT4 on the cell membrane [163], which promotes lactate efflux to the TME, thus decreasing the extracellular pH [40]. In a different context, butyrate was shown to stimulate the function of the gene promoter of the MCT1 transporter, which facilitates the import of lactate to the tumor cells [39], in colorectal adenocarcinoma cell lines [164], decreasing the levels of lactate in the TME. Consequently, it is plausible that intratumoral microbiome-derived SCFAs could exert opposite effects on the regulation of lactate shuttle in the TME, depending on the tumor type, and differentially modulate the tumor pH.

As more and more microorganisms are identified in human tumors, it is important to understand the mechanisms that they employ to adapt the environmental conditions of the TME, and specifically the pH conditions. For instance, the species *Fusobacterium nucleatum*, which predominates in different tumor types, has an optimal growth pH 7.4 [165], so one wonders how it is so widespread in the acidic TME. Moreover, more attention should be given to the potential effects of intratumoral microbiota in modulating the tumor pH, either directly via their secondary metabolites or indirectly by altering the expression of lactate and/or proton transporters on the tumor cell membranes.

A list of identified bacterial species and their effects in hypoxic and acidic TME compartments in different cancer types are included in Supplementary Table S1B. 

## 6. Crosstalk between the Intratumoral Microbiome and the Hypoxic TME 

Given that the hypoxic TME is expected to be inhabited by anaerobic microbial species, anaerobic bacterial genera, such as *Bifidobacterium* and *Clostridium*, accumulate or germinate, respectively, in hypoxic tumor regions [166,167]. Accordingly, in human and murine breast cancer models, an increase in facultative anaerobic species and a concomitant decrease of anaerobes was monitored within the tumor tissue [75]. Interestingly, the same study provided clear evidence that the aerobic bacteria accumulate in lung metastatic tissues, whereas the facultative anaerobe population is diminished, thus highlighting the different oxygen levels between the primary breast and metastatic lung tumors [75]. A cohort analysis of patients with HNSCC further revealed that specific anaerobes are significantly associated with hypoxia scores in distinct tumor regions and types. For instance, *Pseudomonas* was correlated with hypoxia score in oral cavity tumors, while *Actinomyces* and *Sulfurimonas* were correlated with hypoxia score in oropharynx tumors [91]. 

At a molecular level, the intratumoral bacterial load in lung cancer tissues was positively correlated with the gene expression of *HIF-1A* and *VEGF-A* in cancer cells, whose encoded proteins promote tumor hypoxia and angiogenesis, respectively, thus leading to cancer progression [74]. Similarly, the intratumoral injection of *Neospora caninum* tachyzoites in mice bearing B16.F10 melanoma tumors displayed a trend towards elevated *HIF-1α* gene expression in the tumor site [94]. The expression of VEGF-A in breast tumor tissues was also positively correlated with the intratumoral presence of *Pelomonas*, whereas it was negatively correlated with the intratumoral abundance of members of the *Bradyrhizobium* genus [144]. Additionally, the presence of Parapoxvirus signatures in TNBC tissues has been reported to be accompanied by expression of the viral homologs of human *VEGF-A*, *VEGF-E*, which induce the survival and metabolic adaptation of breast tumor cells [90]. Similarly, high intratumoral microbial abundance in neuroblastoma tumors was associated with elevated levels of VEGF within the TME and a negative disease outcome [168] (all summarized in Table S1B). 

From a mechanistic point of view, microbial metabolites have been assumed to play a dual role in HIF-1 stabilization and neovascularization in the hypoxic tumor milieu. For instance, in a *Clostridium difficile*-induced colitis model, butyrate mediated the stabilization of HIF-1 expression in intestinal epithelial cells [169]. Furthermore, the elimination of bacteria from the gut lumen results in a lower oxygen consumption by the luminal epithelial cells, presumably due to the decreased production of SCFAs, including butyrate, which is known to promote oxygen consumption and HIF-1α stabilization in these cells [170]. On the other hand, the incubation of CRC cells with butyrate has been reported to diminish significantly the secreted VEGF levels, which is attributed to the decreased HIF-1α nuclear translocation and DNA-binding activity, thus driving defective angiogenesis [171]. Moreover, along with butyrate, additional microbial metabolites that are also detected in CRC TME, such as propionate and reuterin, were able to hinder the HIF-2α protein expression and functionality in the same CRC model via various mechanisms, including obstruction of its heterodimerization [172,173]. Given these contradictory findings, the actual role of butyrate on the hypoxic niche of the colorectal TME remains to be clearly elucidated. 

In a different context, the metabolite biliverdin, synthesized by *Enterococcus faecalis* within CRC tissues [174], induces the expression of VEGF-A in vitro and *in vivo*, which in turn promotes angiogenesis and tumor progression [175]. Lactate, another metabolite that is also produced by intratumoral bacteria, was able to activate HIF-1α and its downstream pro-angiogenic factor VEGFR2 in normoxic endothelial cells, while the blockade of the MCT1 lactate transporter prevented tumor angiogenesis in lung and hepatic tumor mouse models [176]. Overall, the above findings reinforce the critical impact of bacterial-derived metabolites, including SCFAs, in sustaining the hypoxic TME and favoring tumor cell survival and proliferation. The effects of microbiome-derived metabolites on distinct TME compartments, including the hypoxic TME, are summarized in Supplementary Table S1C.

Given the numerous examples of positive or negative correlations of intratumoral microbiota or the metabolites produced by them with the expression of HIF or VEGF proteins, more studies designated to elucidate the role of intratumoral microbiome in sustaining the hypoxic niche and their association with tumor angiogenesis is warranted.

## 7. Crosstalk between the Intratumoral Microbiome and the Metabolic TME 

An emerging concept connects the dysregulated metabolome of tumors with the intratumoral microbial composition (Supplementary Table S1D). For example, in gastric tumors, multiple distinct metabolites spanning from fatty acid to amino acid metabolic pathways were significantly correlated with a number of different intratumoral bacterial genera [177]. Positive correlations between the abundance of *Lactobacillus* and *Muribaculaceae* members and altered metabolites belonging to glutathione, glucose or amino acid metabolic pathways have also been described in distal or proximal gastric tumors [178], while *Prevotella*, *Acinetobacter* and *Streptococcus* [177,178], have the inherent capacity to synthesize lipid precursor molecules, including diacylglycerols and phosphatidylethanolamines [179], in the same tumor type. All the above findings imply that intratumoral microbiome could either influence the biosynthetic pathways of several metabolites in the TME or contribute to their biosynthesis and/or degradation.

The effect of *Escherichia coli* secretome on the metabolic profile of MDA-MB-231 breast cancer cells was remarkably pronounced, as several lipid, carbohydrate and amino acid metabolic pathways were dysregulated in the presence of *E. coli* metabolites [180]. Given the abundance of *E. coli* in breast cancer tissues [181], it is presumed that its intratumoral presence might affect tumor cell metabolism in a similar manner. Additionally, in TNBC tissues, the intratumoral phylum Tenericutes was positively associated with the levels of sphingomyelin and ceramide in the TME. Apart from this, several intratumoral bacterial phyla, including Firmicutes, Bacteroidetes and the highly abundant Proteobacteria, were significantly correlated with the load of lipid metabolites within the tumor, providing evidence that intratumoral microbiome modulates the metabolic landscape of the TME in TNBC [182]. Another prompt example of how the intratumoral microbiome can affect the metabolic TME was illustrated by the intratumoral administration of the non-pathogenic bacterial strain *Escherichia coli* Nissle 1917, engineered to convert ammonia to the immunostimulatory L-arginine, in MC38 mouse models. This engineered strain accumulated within the tumors and elevated the levels of L-arginine in the TME, while it delivered synergistic action with PD-L1 blockade to shrink the tumor volume [183]. 

Furthermore, the oral administration of *Clostridium butyricum* in orthotopic mouse models of PDAC led to increased tumor colonization by *C. butyricum* and decreased tumor weight, while the primary metabolite of *C. butyricum*, butyrate, drove the accumulation of ROS, lipid droplets and triglycerides along with the downregulation of superoxide dismutase 2 in a pancreatic cancer cell line, ultimately inflicting metabolic disruption and cell death of the cancer cells and thus providing a putative mechanism of how *C. butyricum* acts in the pancreatic TME to promote tumor regression [107]. Similarly, *Lactobacillus reuteri* and its main metabolite, reuterin, are scarce in human and murine colorectal tumor tissues in comparison with healthy colon tissues; additionally, the supplementation of colon tumor-bearing mice with *L. reuteri* inhibits tumor growth in a reuterin-dependent manner, as it is reported that reuterin induces oxidative stress in colon cancer cells by protein oxidation and impaired ribosomal biogenesis, impeding protein translation and cancer cell growth [172]. In this context, mice bearing Lewis lung carcinoma (LLC) tumors displayed high intratumoral bacterial levels and altered microbial composition within the tumor after oral gavage with *Akkermansia muciniphila*, while a downregulation in lactic acid production with a concurrent decrease in the expression of the enzyme lactic dehydrogenase A were also observed in the TME [69]. Tumor-generated lactic acid and products of glutamine catabolism, such as glutamic and succinic acid, as well as nucleotide precursors, including AMP, ADP, UMP and GMP, are thought to promote metabolic reprogramming in cancer cells, which in turn entails developmental advantages and increased cell resistance to hypoxic conditions, while lactic acid can also act as an immunosuppressive metabolite [184]. In accordance with this notion, the levels of nucleotide biosynthesis and glutamine metabolites in the LLC tumors were also negatively impacted by the *A. muciniphila* oral gavage, suggesting that *A. muciniphila* hinders tumor progression by the metabolic modulation of the TME [69]. Given that in the control LLC mouse model without oral gavage, the intratumoral abundance of bacteria belonging to *Acidobacteriales* and *Acidobacteriaceae* families was positively associated with the presence of lactic acid in the tumor milieu, it is clearly denoted that specific intratumoral microbe composition might have opposing roles on shaping a tumor metabolic profile that could be either permissive or suppressive for cancer progression [69]. 

Noteworthy, intratumoral microbiome cannot only impact the metabolic components of the TME but its own metabolism can also influence cancer treatment outcomes. For instance, the intratumoral microbiome composition and diversity is suggested to mediate resistance to chemotherapeutic drugs, as in the case of bacteria belonging to the Gammaproteobacteria class, which are present in certain tumors and able to metabolize gemcitabine into its inactive form by the long isoform of the bacterial enzyme cytidine deaminase [72]. Additionally, intratumoral bacterial species like *Escherichia coli* isolated by human colorectal cancer tissues were also capable of depleting the chemotherapeutic drug 5-fluorouracil (5-FU) and abrogating its toxicity against colorectal tumor cells or other 5-FU-sensitive microbes, such as *Fusobacterium nucleatum* [185]. In cervical cancer, tumor-resident lactate-producing *Lactobacillus iners* was associated with reduced patient response to chemoradiation, mediated by augmented lactate production in the TME and tumor metabolic remodeling, which in turn encompasses upregulated glycolysis, TCA cycle and DNA synthesis [186]. These effects of the tumor-associated *L. iners* strains were presumably attributed to the acquisition of an additional gene, *lacG*, ensuing the production of galactose and its subsequent conversion to lactate, thus exacerbating the lactate levels in the TME [186]. In a different context, the presence of *Paraburkholderia fungorum* within tumors of mice engrafted with cholangiocarcinoma cells drove the upregulation of specific metabolites in the TME, which were related to metabolic pathways of alanine, aspartate and glutamate [82]. Several intratumoral bacterial genera were further correlated with certain metabolites present in patient-derived cholangiocarcinoma tissues treated with different doses of gemcitabine or cisplatin, thus implying a connection between the intratumoral microbiome and the metabolic modulation of the TME to promote drug responses [187]. All the findings from the above studies highlight the role of intratumoral microbiome in the modulation of cancer chemoresistance and warrant new insights for further investigation. 

Last but not least, it is possible that the crosstalk developed among the intratumoral microbiome, the tumor or immune cells within the TME and the altered metabolic features of the latter, such as oxygen consumption or nitrate production, dictate the microbial signature of the TME, in a manner resembling the interplay between colonocytes and microbiome in the gut [188]. 

One question contingent on the prevalence of microorganisms in human tumors is whether they contribute to the reservoirs of secondary metabolites found in the TME that were considered to originate from other sources, such as lactate and SCFAs, and to what extent. It is important to gain more insights into how the tumor metabolism is interconnected with the intratumoral microbiome metabolism by more vigorous investigation on how microbial metabolites affect the metabolic niche of the TME in different tumor types. Another prominent question that arises based on studies describing a connection between intratumoral microbiome metabolism and drug responses in the TME is whether this phenomenon could be more generalized in microorganism or tumor contexts. Apart from the intratumoral microbiome metabolism itself, it is also worth investigating if tumor-resident microbiota could shape the metabolic TME in a certain way, rendering it more permissive or resistant to chemotherapeutic treatments.

## 8. Crosstalk between the Intratumoral Microbiome and the Mechanical TME 

The effects of the intratumoral microbiome composition on the remodeling of the mechanical TME compartment have recently gained enough attention (Supplementary Table S1E). Noteworthy, several bacteria that inhabit tumor tissues have the inherent ability of producing ECM remodeling enzymes [189], such as hyaluronidases (Gram-positive bacteria) [17], collagenases (*Pseudomonas aeruginosa*) [96], metalloproteinases (*Bacteroides fragilis*) [190] and elastases (*Listeria monocytogenes*) [142], which might directly modulate the tumor stroma. For instance, the load and diversity of the intratumoral microbial species present in muscle invasive bladder carcinoma (MIBC) display positive or negative correlations with the expression profiles of numerous ECM-related genes in MIBC tissues, thus indicating a potential role of intratumoral microbiome in shaping the mechanical features of the TME [191]. In the same tumor model, the intratumoral abundance of *E. coli* str. K-12 substr. MG1655 and the butyrate-producing bacterium SM4/1 were negatively associated with E-cadherin expression, whereas the presence of *Actinosynnema mirum DSM 43827* and *Burkholderia ambifaria AMMD* revealed inverse and positive correlations with COL26A1 and elastin expression levels, respectively [191]. 

On the other hand, patient-derived CRC tissues were characterized by decreased concentrations of *Bifidobacterium adolescentis*. When this strain was used as a treatment modality in CRC mouse models, a distinct subpopulation of CAFs, overexpressing the marker CD143, was induced in the TME. This induction was mediated by Wnt/β-catenin signaling activation and eventually resulted in tumor suppression [192]. Another study suggests that the intratumoral accumulation of *Actinomyces* within the CAFs of the colorectal TME, as well as that its loads are significantly correlated with the αSMA+ tumor stromal cells, therefore underpinning a direct interaction between intratumoral microbiome and tumor stroma [193]. Furthermore, the microbial metabolite, butyrate, was found to severely affect the capacity of colon carcinoma cell lines to attach to type I and IV collagen-coated surfaces through downregulation of α_2_β_1_ integrin expression [194], thus providing insights on how butyrate-producing bacteria could modify the interactions between tumor cells and ECM in the colon TME. Interestingly, the high prevalence of the bacterium *Fusobacterium nucleatum* in the colorectal TME employs a mechanism of hijacking the tumor cell E-cadherin/β-catenin axis through direct binding of E-cadherin to its FadA adhesin in order to promote CRC cell invasion [195]. An alternative mechanism that enables *F. nucleatum* colonization and enrichment in tumor tissues has been described in breast cancer tumors [196], and involves the overexpression of the disaccharide moiety Gal-GalNAc in human tumor tissues, which is bound by the *F. nucleatum*-expressing lectin Fap2 [71].

Moreover, the abundance of *Porphyromonas gingivalis* in esophageal cancer milieu has been associated with acceleration of tumor growth, through a mechanism that involves induction of the MMP-9 and consequent downregulation of E-cadherin expression, thus skewing the tumor cells towards a mesenchymal phenotype and disrupting their interactions with the surrounding ECM [197]. Similarly, the infection of human stomach fibroblasts with *Helicobacter pylori*, a pathogen commonly found in gastric tumor tissues, promotes the expression of VCAM1 via activation of JAK/STAT1 signaling. A proposed mechanism of *H. pylori*-mediated gastric cancer progression is through VCAM1 upregulation in gastric CAFs, which in turn potentiates the invasiveness of gastric tumor cells via binding to their αVβ5/1 integrins [198]. Other evidence suggesting the implication of this pathogen in reshaping the malignant tumor stroma has derived after incubation of normal rat fibroblasts with *H. pylori*, which was able to promote the differentiation to CAFs with cell phenotypes overexpressing α-SMA, type I and III collagens, as well as several proinflammatory markers [199]. The complex crosstalk between the intratumoral microbiome and the mechanical TME is further supported by findings indicating that the deletion of the type I collagen homotrimer in murine pancreatic tumor cells results in intratumoral microbial signature reshaping, which becomes highly enriched in *Campylobacterales* and declined in *Bacteroidales* populations, which are overall involved in T cell recruitment in the TME [200]. 

There are still many gaps in understanding the crosstalk of the intratumoral microbiome and the mechanical TME. Firstly, more studies in preclinical models are needed to shed light on the roles of more microorganisms in modifying the tumor stroma, along with the underlying mechanisms. An interesting aspect of this would be the interrogation of the capacity of certain intratumoral microbiome to directly modulate the tumor stroma, by generating ECM remodeling enzymes. Vice versa, it remains obscure whether alterations in the stroma of solid tumors make them more permissive to certain types of microbiota and ultimately affect their microbial composition. Moreover, the finding that intratumoral microbes can invade CAFs [193] fuels the speculation about their impact on CAFs properties and consequently on the mechanical TME.

## 9. Crosstalk between the Intratumoral Microbiome and the Innervated TME 

There is increasing evidence of constant crosstalk between the intratumoral microbiome and the innervated tumor niche, mainly affecting the so called perineural invasion, a rare and under-recognized route of metastatic spread that refers to the invasion of neoplastic cells to the space surrounding a nerve (Supplementary Table S1E). The load and limited richness of specific microbial species have been significantly associated with advanced perineural invasion, as observed in the case of OSCC and rectal cancer TMEs [190,201]. Similarly, in HNSCC tissue samples, the reduced presence of the intratumoral fungal species *Aspergillus flavus*, *Coccidioides immitis RS* and *Gaeumannomyces tritici R3-111a-1* was correlated with decreased perineural invasion [202]. Additionally, the intratumoral accumulation of *Morchella esculenta* was associated with lack of perineural invasion in HPV-negative HNSCC tissues [202]. Moreover, the high intratumoral abundance of the fungal genus *Solicoccozyma* or the *Solicoccozyma aeria* species was correlated with the absence of perineural invasion in human gastric tumors [203]. 

Apart from their impact on perineural invasion of tumors, some bacterial species that inhabit human tumors are capable of producing certain neurotransmitters that are known to affect the properties and the functionality of TME cell populations, including tumor, immune and endothelial cells [204]. Characteristic examples are the γ-aminobutyric acid (GABA) synthesized by *Lactobacillus brevis* and *Bifidobacterium adolescentis* [205] found in gastric and colorectal cancer, respectively [206,207], and dopamine and serotonin, produced by *Klebsiella pneumoniae* or the *E. coli* strain K-12 [208,209], which inhabit pancreatic and HNSCC tumors [17,202]. Interestingly, a link between the intratumoral abundance of *Delftia acidovorans SPH-1* and the dysregulated gene expression of the brain-derived neurotrophic factor (BDNF) was described in prostate cancer tissues, highlighting the possibility that intratumoral microbiome could also alter the expression of neurotrophins in cancer cells [142].

Based on the above, it is plausible to reflect on whether the intratumoral microbiome could also possess a role in guiding tumor innervation, apart from its well-established impact on perineural invasion. This could be possibly achieved through direct secretion of signaling factors, such as neurotransmitters or neuropeptides, or modulation of the secretion of such factors by other cell types in the TME. Undoubtedly, the relationship between the intratumoral microbiome and tumor innervation is an understudied area, requiring significantly more research to gain a deeper understanding.

**Figure 3:**
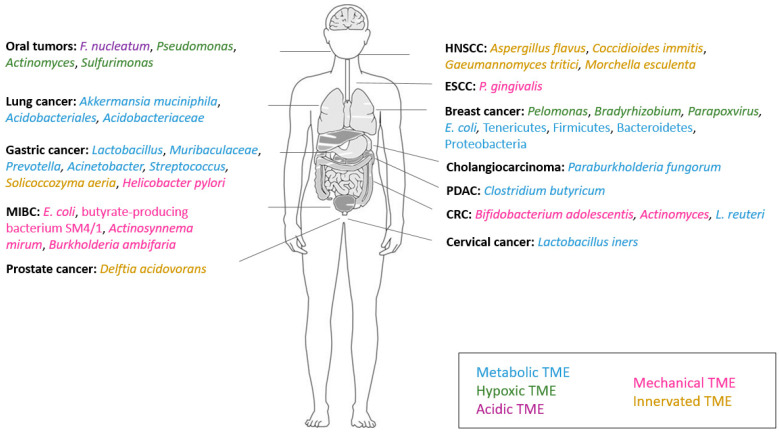
Graphic summary of the intratumoral phyla, genera or species associated with the metabolic (blue), hypoxic (green), acidic (purple), mechanical (pink) and innervated (orange) sub-microenvironments of the TME, in different tumor types. More details on their role on the respective sub-microenvironment can be found in the text.

The intratumoral microbiota associated with the acidic, hypoxic, metabolic, mechanical and innervated compartments of the TME are outlined in Figure 3.

## 10. Perspectives on Microbiome-Mediated, Multifaceted and Multileveled Effects on the TME Landscape Remodeling—Therapeutic Interventions

Due to the extensive intertwinement among the distinct TME compartments, it is not surprising that the intratumoral microbiome can simultaneously exert its multifaceted effects on multiple TME segments. For instance, the depletion of bacteria from pre-metastatic niches in the liver of mice bearing colon tumors diminished liver infiltration by innate immune cell populations along with the expression of proinflammatory cytokines and chemokines, while it simultaneously impaired the ECM deposition by downregulating the expression of metalloproteinases and collagens [70]. The intratumoral accumulation of the microbial metabolite 3-IAA [100,110] has been recognized as an agent promoting neutrophil degranulation and myeloperoxidase release when administered in combination with the chemotherapeutic scheme FIRINOX (5-FU, irinotecan and oxaliplatin) to mice bearing PDAC tumors. The myeloperoxidase release along with the presence of 3-IAA and FIRINOX subsequently act by triggering ROS accumulation in PDAC cells, ultimately dampening their metabolic fitness and proliferation [111]. This is an exquisite example of how microbiome-derived metabolites could simultaneously impact different TME compartments, as in this case the immune and metabolic TME niches. 

Moreover, compared to virus-enriched HCC tissues, bacteria-enriched tumors were characterized by elevated amino acid metabolism and CD163+ (M2) macrophage presence, thus underscoring the role of intratumoral bacteria as mediators between the immune and metabolic microenvironments in the HCC [210]. Similarly, the intratumoral presence of *Fusobacterium animalis* in mesenchymal colorectal cancer subtypes was associated with both upregulated collagen synthesis and cytokine signaling pathways, thus exerting a dual role in reshaping the mechanical and immune niches of the colorectal TME [211]. Accordingly, in a murine orthotopic breast cancer model, the intratumoral colonization of *Fusobacterium nucleatum* exacerbated tumor progression by reducing TME infiltration by CD4+ and CD8+ T cells, while the incubation of the injected AT3 breast cancer cells with *F. nucleatum* triggered the overexpression of MMP-9. These findings highlight the purported, dual role of intratumoral *F. nucleatum* on both the immune and mechanical microenvironments within the breast cancer TME [196]. 

In this context, the intratumoral injection of attenuated *Staphylococcus aureus* bioparticles in mouse models of LLC resulted in the recruitment of monocytes, dendritic cells and CD3+ T cells in the TME but most importantly favored the infiltration of a population of neutrophils with upregulated Gr-1 and CD11b expression and downregulated CD62L and CXCR2 levels. This microbial treatment stimulated the neutrophil motility, potentiated their survival in the TME and boosted the CD8+ T effector function in the tumor tissue, ultimately inhibiting tumor growth. Concomitantly, following the *S. aureus* injection, the expression levels of VEGF were decreased in neutrophils, while the expression of the inducible nitric oxide synthase, which entails the release of ROS, was upregulated in the infiltrating neutrophils, thus highlighting the effects of the intratumoral *S. aureus* bioparticles on the immune, metabolic and hypoxic compartments of the TME [212]. Moreover, a cohort analysis in patients with metastatic cervical cancer revealed that an intratumoral microbiome signature comprising *Robiginitomaculum*, *Microbispora*, *Klebsiella* and *Micromonospora* was correlated with increased expression of the surface receptors PD-1 and CTLA-4, and the egl-9 family hypoxia-inducible factor 1 (EGLN1), as well as with worse disease prognosis. Interestingly, EGLN1 expression is negatively correlated with CD8+ T cell infiltration within the TME, while positively associated with accumulation of CD4+ resting memory T cells, M0 macrophages and activated mast cells, which partly explains the negative predictive value of this microbiome subset on the disease outcome, via remodeling the hypoxic and immune TME niches [213]. Noteworthy, the potential role of microbiome as a multileveled regulator of distinct TME segments is further illustrated by a recent study showing that gut microbiota regulate the expression of the addressin MAdCAM-1 in intestinal endothelial and lymphoid tissues, thus controlling the egress of enterotropic α4β7+/IL-17+ regulatory T cells to tumor tissues by modulating the interaction between MAdCAM-1 and α4β7 [214]. It is likely that such checkpoint mechanisms could also be in place within the TME, where the intratumoral microbiome could act locally to modify disparate TME niches and impact the tumor immunosurveillance.

Given the prominent role of the intratumoral microbiome in modulating different TME components and impacting the cancer progression, thoughts about its putative therapeutic targeting in cancer are constantly gaining ground. A number of preclinical studies in cancer mouse models directly point out the value of antibiotic administration in tumor regression [87,137,155,196], while others have described the tumor-promoting role of certain intratumoral microbes, thus implying that their elimination could benefit the disease outcomes [61]. Nonetheless, the use of antibiotics in the clinical setting for the treatment of cancer remains controversial, as it has been shown to interfere with the efficacy of immune checkpoint-based immunotherapy, resulting in significantly reduced PFS in multiple types of malignancies [128,152,215]. Ongoing clinical trials attempt to decipher the potential value of antibiotics in cancer therapy, such as NCT05777603, which evaluates the efficacy of aerosolized antibiotics in combination with pembrolizumab in non-small cell lung cancer (NSCLC). 

On the other hand, the existence of specific intratumoral microbes has been shown to suppress tumor growth by remodeling the immune profile within the TME. Characteristic examples are the presence of *Lactobacillus reuteri* and *Bifidobacterium* bacteria in melanoma [110] and CRC tumors [119], which potentiate the action of immune checkpoint inhibitors and highlight the possibility of probiotic administration as an adjuvant to immunotherapy. Thus far, several clinical trials (NCT03705442, NCT05032014) have investigated the effectiveness of probiotics in cancer treatment but without conclusive results. Currently, a phase IV clinical trial studying the role of probiotic supplementation in concert with immunotherapy against urothelial bladder carcinoma (NCT05220124) is under way. 

As already mentioned, there are many instances of harnessing microbes, especially bacteria, as vehicles for the delivery of specific products in the TME, such as chemokines [127], cytokines [126], tumor antigens [60] and metabolites [183], that can potentially modulate the TME immune landscape and enhance the effects of immunotherapy. Remarkably, findings of such preclinical studies led to the first human phase I/II clinical trial, which evaluates the action of a genetically engineered, attenuated *Yersinia enterocolitica* strain in patients with advanced solid malignancies (NCT05120596) after intratumoral or intravenous administration, with or without pembrolizumab.

## 11. Challenges and Limitations in Studying Microbiome–TME Interactions

The intratumoral microbiome can be characterized as low-biomass microbial life, as the microbial communities within the TME are underrepresented. Consequently, studying this low-biomass microbiome faces several conceptual and methodological challenges that may interfere with the overall conclusions regarding the impact of certain microorganisms on distinct tumor microenvironments, as well as on cancer development, progression and response to treatment.

A major conceptual and methodological hurdle is defining the significance and impact of the detected microbiomes within the tumors, as well as the sensitivity and specificity of the methods used for microbiome detection. The presence of microbial DNA does not substantiate active microbial interference with the TME, while the conventional microbial detection methods might not be sufficiently sensitive to reveal the diversity of the microbial life present in the TME, thus necessitating the use of more advanced detection technologies. In addition, elucidating underlying mechanisms by which microbiome may interact with cancer cells and influence their properties is crucial in understanding the microbial impact in the TME. Contamination concerns at each step of sample collection, processing and analysis have been also highlighted as major methodological and conceptual obstacles, as they can adulterate the profile of the true microbial inhabitants within the TME and eventually our conclusions. Moreover, given the cancer heterogeneity, both within a single tumor and between different patients, microbial colonization and activity may vary in their interaction patterns with the TME. Therefore, the sample’s integrity and quantity are of high importance as they can determine the comprehensiveness of the microbiome analysis. Lastly, interpreting data and validating findings derived by low-biomass intratumoral microbiomes are equally challenging requiring advanced bioinformatic approaches and high reproducibility across different studies and cohorts.

Acknowledging all the aforementioned limitations in a conceptual and methodological context, research conclusions regarding the potential impacts of microbiome–tumor interactions may be challenged by (i) data bias due to contamination and detection issues, (ii) misinterpretation of results concerning the role and significance of certain microbiome communities within the TME, and (iii) hindered mechanistic insights through which microbes may affect tumor biology and ultimately the clinical translation of findings and their applications. 

Another concern that should be taken under serious consideration in translational research studies on intratumoral microbiome–TME interactions is the difficulties of extrapolating results from laboratory mouse models to humans. These challenges mainly stem from several key factors that can profoundly impact the interpretation and applicability of basic and preclinical research findings. For instance, the two species pose substantial differences in (i) physiology (e.g., metabolic variations may determine the nutrient substrate that supports the growth and action of specific microbiome communities, thus influencing microbiome composition, behavior and interaction with cancer cells), (ii) anatomy and system actions (e.g., size of organs and tissues may reflect differences in TME structure and microbiome composition, as well as in immune system functions and crosstalk with intratumoral microbiome), (iii) exposing environments (e.g., exposure of laboratory animals to controlled laboratory conditions usually minimize or eliminate exposure to pathogens, a fact that may interfere with the intratumoral microbiome composition), and finally in (iv) dietary habits, as humans versus experimental mice follow a less standardized and highly varied diet leading to discrepancies in how microbial species develop and sustain themselves within tumors. Overall, to overcome these obstacles and advance our understanding and translation of intratumoral microbiome research, there must be increased focus on human studies and the development of models that better mimic human conditions.

## 12. Conclusions

In a nutshell, the intratumoral microbiome is critical in shaping the features of distinct, specialized tumor microenvironments, while it emerges as an orchestrator of tumor responses, by its multileveled actions in the different TME niches. Its effects on the TME compartments ultimately impact the tumor development and disease outcomes in a contradictory manner, depending on the intratumoral microbial composition and abundance, as well as on the tumor type. Further studies are needed to clearly elucidate the functional characteristics of the complex microbiome–TME interplay and inform therapeutic strategies for efficient intratumoral microbiome targeting and manipulation in cancer patients. However, addressing the various conceptual and methodological issues, as well as the limitations of experimental models analyzed above, is critical for accurately interpreting the interactions between low-biomass microbiomes and the TME, and for translating these findings into clinical practice. These obstacles can be overcome by adopting improved intratumoral microbiome detection technologies, implementing controls to ensure detection accuracy, and promoting reproducibility through the standardization and validation of methods used across multiple human studies and cohorts. 

## Data Availability

No new data were created or analyzed in this study. Data sharing is not applicable to this article.

## Supplementary Materials

The following supporting information can be downloaded at: https://www.mdpi.com/article/10.3390/cells13151279/s1, Table S1A: Identified intratumoral microorganisms and their effects on immune TME compartment in different types of malignancies, Table S1B: Identified intratumoral microorganisms and their effects on Hypoxic & Acidic TME compartments in different types of malignancies, Table S1C: Identified intratumoral microbiome-derived metabolites and their effects on distinct TME compartments in different types of malignancies, Table S1D: Identified intratumoral microorganisms and their effects on Metabolic TME compartment in different types of malignancies, Table S1E: Identified intratumoral microorganisms and their effects on Mechanical & Innervated TME compartments in different types of malignancies.
