# Supplementary material for: Intratumoral Microbiome: Foe or Friend in Reshaping the Tumor Microenvironment Landscape?
Source: Cells. 2024 Jul 30;13(15):1279. doi: 10.3390/cells13151279 (PMC11312414; doi:10.3390/cells13151279)
Supplement: Supplementary file 1 [file cells-13-01279-s001.zip › cells-3069626-supplementary.pdf]

Table S1A. Identified intratumoral microorganisms and their effects on immune TME compartment in different types of malignancies

Microorganism	Tumor type	Immune TME	Reference
Blautia, Faecalibacterium, Faecalitea	CRC	↑CD3 infiltration	[113]
Sachharopolyspora, Pseudoxanthomonas, Streptomyces	PDAC	↑CD8 infiltration, granzyme B production	[19]
Lachnoclostridium	Melanoma	↑CD8 infiltration, elevated CXCL9, CXCL10 and CCL5	[114]
Fusobacterium nucleatum	OSCC	↓B cells, CD4+ T helper cells & M2 macrophages	[83]
Streptococcus	ESCC	↑ CD8+, Granzyme B+ cells	[115]
Clostridium novyi spores	Refractory solid tumors	↑CD8+, Tregs, MDSCs	[116]
PVSRIRO	Melanoma	↑CD4+, CD8+, NK cells, elevated IFNγ and granzyme B	[117]
Neospora caninum	Melanoma	↑ CD8+, CD68+ cells, ↑ Th1 cytokines	[94]
Firmicutes, Proteobacteria	GBM	↑Activation of TIL-derived CD4+ clones, ↑pro-inflammatory cytokines & chemokines	[118]
Lactobacillus reuteri (effect mediated by I3A)	Melanoma	↑Tc1 phenotype, ↑ Ahr signaling in CD8+ cells, ↑ IFNγ	[110]
Bifidobacterium	CRC	↑STING-mediated type I IFN signaling in dendritic cells	[119]
Lactobacillus reuteri (effect mediated by SCFA)	HCC	↓IL17A in hepatic ILC3	[121]
Malassezia	PDAC	↑Mannose-binding lectin/C3 complement cascade	[122]
Engineered *Salmonella* strain VNP20009	Melanoma	↑Activation of dendritic cells	[123]
Engineered *E. coli* Nissle 1917 strain	Breast cancer	↑CD4+, ↑ IFNγ, TNFα	[124]
Engineered *E. coli* strain	Breast cancer	↑MHC-II, CD86 on dendritic cells ↑ CD8+ IFNγ+ T cells, ↓ Tregs	[125]
Engineered *E. coli* strain MG1655	Renal cancer /CRC	↑IFNγ, IL-12	[126]
Engineered *E. coli* strain Nissle 1917	B-cell lymphoma	↑IFNγ & Granzyme B secretion by CD4+ & CD8+ T cells	[127]
Enterococcus faecalis, Lactobacillus johnsonii, Escherichia coli	Melanoma	↑ MHC-II, CD40, CD80, CD86 on dendritic cells	[128]
Firmicutes, Bacteroidetes	CRC	↓IL-17A, CCL20	[129]
Achromobacter deleyi, Microcella alkaliphila	Ovarian cancer	↑M1 macrophages	[130]
Devosia sp. LEGU1, *Ancylobacter pratisalsi, Acinetobacter seifertii*	Ovarian cancer	↓M1 macrophages	[130]
Bacillus, Paenibacillus (effect mediated by TMAO)	PDAC	Skewing TAMs, MDSCs, CD4+, CD8+, dendritic cells to immunostimulatory phenotypes	[131]

Alistipes	CRC	↑CD8 naïve & memory T cells ↓ macropahges, NK, MAIT cells	[134]
Parvimonas, Bilophila	CRC	↓NK & MAIT cells	[134]
F. nucleatum	CRC	↓CD3+ infiltration, ↑TAMs, MDSCs, dendritic cells, ↑CD163+ ↓ Foxp3+ T cells	[61,135, 136]
Clostridiales	CRC	↓CD8+ cells	[138]
Porphyromonas gingivalis	CRC	↑CD11b+, macrophages, dendritic cells ↑ TNFα, IL-6 and IL-1β	[139]
Porphyromonas gingivalis	PDAC	↓CD8+ cells ↑ neutrophils ↑ CXCL1, CXCL2, CXCR2, neutrophil-derived elastase	[62]
Acidovorax ebreus TPSY	PDAC	↓CD8+ cells, activated memory T cells, M2 macrophages	[140]
Algibacter, *Epilithonimonas*	Melanoma	↓CD8+ cells, CXCL9, CXCL10 and CCL5	[114]
Pseudomonas putida strain KT2440	LUSC	↓naïve B cells and activated dendritic cells	[143]
Rothia dentocariosa ATCC 17931	LUSC	↓naïve B cells, resting mast cells, M1 and M2 macrophages	[143]
Thermostaphylospora chromogena	LUSC	↓naïve B cells, resting CD4+, NK, activated mast cells	[143]
Actinobacter	Breast cancer	↑CD8+ T cells	[144]
Methylibium	Breast cancer	↓T cell infiltration	[144]
Stenotrophomonas, Acinetobacter, Gemella, Neisseria, Aquabacterium, Haemophilus, Novosphingobium, Streptococcus, Massilia, Gemmiger, *Chryseobacterium, Brevundimonas*	Gastric cancer	↑BDCA2+ plasmacytoid dendritic cells	[146]
Streptococcus, Massilia, Fusobacterium, Oribacterium, Campylobacter, Selenomonas, Dialister, Photobacterium	Gastric cancer	↑Foxp3+ Tregs	[146]
Lactobacillus, F. nucleatum	ESCC	↑PD-L1+ TAMs	[147]
Malassezia	PDAC	↑IL-33, ILC2 cells	[150]
Mycoplasma hyopneumoniae, Citrobacter freundii	PDAC	↑Immune suppression pathways, proinflammatory immune cascades	[140]
Candida albicans	CRC	↑IL-7 production in macrophages, ↑ IL-22 in ILC3 cells	[151]
Lactobacillus murinus, Lactobacillus reuteri (mediated by indoles)	PDAC	↓CD8+, ↑ MDSCs, Ahr activity on TAMs	[153]
Methylobacterium	Gastric cancer	↓CD8+, CD103+ TRM cells	[154]

Table S1B. Identified intratumoral microorganisms and their effects on Hypoxic & Acidic TME compartments in different types of malignancies

Microorganism	Tumor type	Hypoxic TME	Acidic TME	Reference
F. nucleatum	OSCC		↑ GLUT1 on cancer cells, glycolysis, lactate production	[159]
Pseudomonas	Oral cavity tumors	↑Hypoxia score		[91]
Actinomyces, Sulfurimonas	Oropharynx tumors	↑Hypoxia score		[91]
Neospora caninum tachyzoites	Melanoma	↑HIF-1α		[94]
Pelomonas	Breast cancer	↑VEGF-A		[144]
Bradyrhizobium	Breast cancer	↓VEGF-A		[144]
Parapoxvirus	TNBC	↑viral homologs of human VEGF-A, VEGF-E		[90]

Table S1C. Identified intratumoral microbiome-derived metabolites and their effects on distinct TME compartments in different types of malignancies

Microbial metabolite	Tumor type	Hypoxic TME	Acidic TME	Mechanical TME	Reference
Putrescine	GBM		Buffering the intracellular pH of myeloid lineage cells		[161]
Butyrate	Breast Cancer		↑MCT4 on cancer cells, lactate efflux		[163]
Butyrate	CRC	↓VEGF, ↓HIF-1α nuclear translocation	↑MCT1 on cancer cells, lactate influx	↓$\alpha 2\beta 1$ integrin, ↓attachment to type I & IV collagen surfaces	[164, 171, 194]
Butyrate & propionate	CRC	↓HIF-2α			[172, 173]
Reuterin	CRC	↓HIF-2α			[172]
Biliverdin	CRC	↑VEGF-A			[175]

Table S1D. Identified intratumoral microorganisms and their effects on Metabolic TME compartment in different types of malignancies

Microorganism	Tumor type	Metabolic TME	Reference
Lactobacillus, Muribaculaceae	Gastric cancer	Altered glutathione, glucose or amino acid metabolic pathways	[178]
Prevotella, Acinetobacter, Streptococcus	Gastric cancer	Diacylglycerols, phosphatidylethanolamines synthesis	[177-179]
Eshcerichia coli	Breast cancer	Dysregulation of lipid, carbohydrate and amino acid metabolic pathways	[180]
Tenericutes	TNBC	↑Sphingomyelin, ceramide levels	[182]
Firmicutes, Bacteroidetes, Proteobacteria	TNBC	↑Lipid metabolites	[182]
Engineered *E. coli* Nissle 1917	CRC	↑L-arginine	[183]
Clostridium butyricum (effect mediated by butyrate)	PDAC	↑ROS, lipid droplets, triglycerides	[107]
Lactobacillus reuteri (effect mediated by reuterin)	CRC	↑Protein oxidation, impaired ribosomal biogenesis	[172]
Akkermansia muciniphila	Lung cancer	↓Lactate, nucleotide precursors, glutamine metabolites	[69]
Acidobacteriales, Acidobacteriaceae	Lung cancer	↑Lactate	[69]
Gammaproteobacteria	PDAC	↑Gemcitabine metabolism	[72]
Escherichia coli	CRC	↑5-fluorouracil metabolism	[185]
Lactobacillus iners (effect mediated by lactate)	Cervical cancer	↑Glycolysis, TCA cycle, DNA synthesis	[186]
Paraburkholderia fungorum	Cholangio-carcinoma	↑Alanine, aspartate, glutamate metabolites	[82]

Table S1E. Identified intratumoral microorganisms and their effects on Mechanical & Innervated TME compartments in different types of malignancies

Microorganism	Tumor type	Mechanical TME	Innervated TME	Reference
Escherichia coli str. K-12 substr. MG1655, butyrate-producing bacterium SM4/1	MIBC	↓E-cadherin		[191]
Actinosynnema mirum DSM 43827, Burkholderia ambifaria AMMD	MIBC	↓COL26A1, ↑Elastin		[191]
Bifidobacterium adolescentis	CRC	↑CD143+ CAFs		[192]
Actinomyces	CRC	↑αSMA+ stromal cells		[193]
Porphyromonas gingivalis	ESCC	↑MMP-9, ↓ E-cadherin		[197]
Helicobater pylori	Gastric cancer	↑ VCAM-1 in CAFs		[198]
Aspergillus flavus, Coccidioides immitis RS, Gaeumannomyces tritici R3-111a-1, Morchella esculenta	HNSCC		↓Perineural invasion	[202]
Solicoccozyma aeria	Gastric cancer		↓Perineural invasion	[203]
Delftia acidovorans SPH-1	Prostate cancer		BDNF dysregulation	[142]
